# ACTH Receptor (MC2R) Specificity: What Do We Know About Underlying Molecular Mechanisms?

**DOI:** 10.3389/fendo.2017.00013

**Published:** 2017-02-06

**Authors:** Davids Fridmanis, Ance Roga, Janis Klovins

**Affiliations:** ^1^Latvian Biomedical Research and Study Centre, Riga, Latvia

**Keywords:** MC2R, ACTHR, specificity, mutation, mutagenesis, site directed

## Abstract

Coincidentally, the release of this Research Topic in Frontiers in Endocrinology takes place 25 years after the discovery of the adrenocorticotropic hormone receptor (ACTHR) by Mountjoy and colleagues. In subsequent years, following the discovery of other types of mammalian melanocortin receptors (MCRs), ACTHR also became known as melanocortin type 2 receptor (MC2R). At present, five types of MCRs have been reported, all of which share significant sequence similarity at the amino acid level, and all of which specifically bind melanocortins (MCs)—a group of biologically active peptides generated by proteolysis of the proopiomelanocortin precursor. All MCs share an identical –H–F–R–W– pharmacophore sequence. α-Melanocyte-stimulating hormone (α-MSH) and adrenocorticotropic hormone (ACTH) are the most extensively studied MCs and are derived from the same region. Essentially, α-MSH is formed from the first 13 amino acid residues of ACTH. ACTHR is unique among MCRs because it binds one sole ligand—ACTH, which makes it a very attractive research object for molecular pharmacologists. However, much research has failed, and functional studies of this receptor are lagging behind other MCRs. The reason for these difficulties has already been outlined by Mountjoy and colleagues in their publication on ACTHR coding sequence discovery where the Cloudman S91 melanoma cell line was used for receptor expression because it was a “more sensitive assay system.” Subsequent work showed that ACTHR could be successfully expressed only in endogenous MCR-expressing cell lines, since in other cell lines it is retained within the endoplasmic reticulum. The resolution of this methodological problem came in 2005 with the discovery of melanocortin receptor accessory protein, which is required for the formation of functionally active ACTHR. The decade that followed this discovery was filled with exciting research that provided insight into the molecular mechanisms underlying the action of ACTHR. The purpose of this review is to summarize the advances in this fascinating research field.

## Introduction

**Figure 1:**
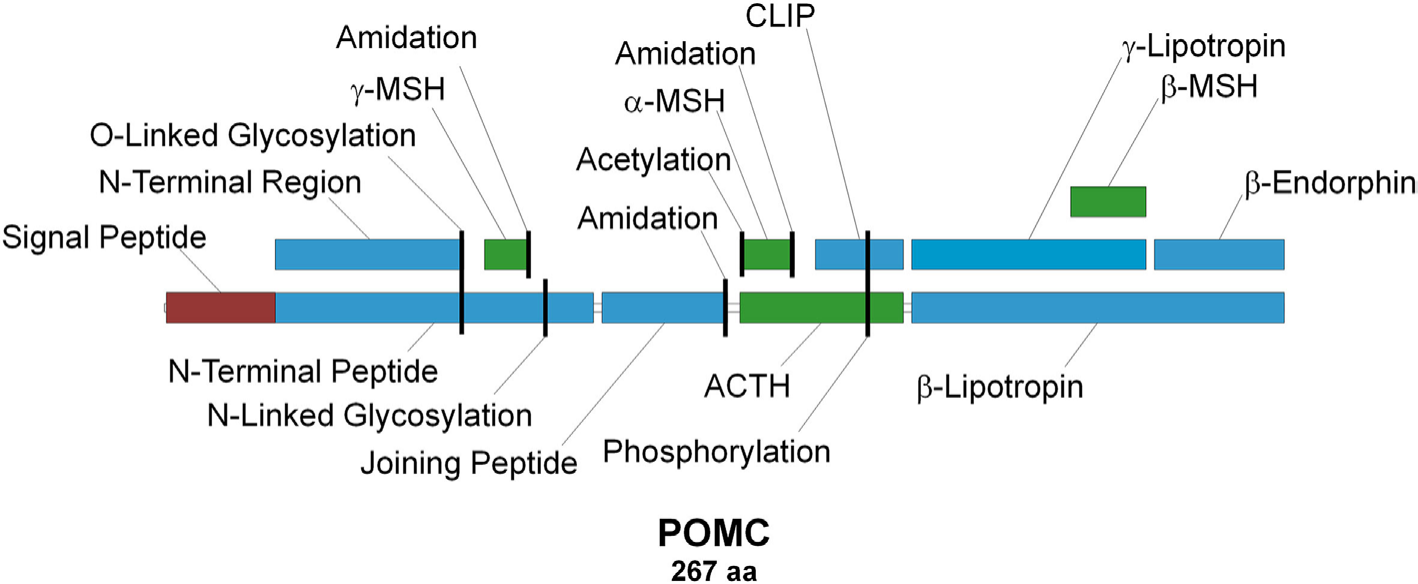
**Schematic representation of the proopiomelanocortin precursor, the products of its proteolysis, and known posttranslational modifications**. Melanocortins are colored green, signal peptide is colored red, and other peptides are colored blue. Posttranslational modification sites are marked with vertical black lines.

**Figure 2:**
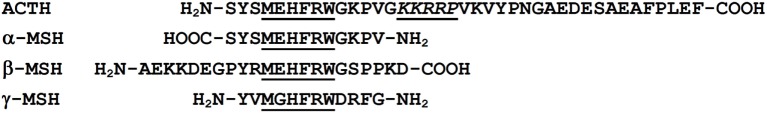
**Sequence alignment of melanocortin (MC) peptides**. All MCs share the conserved –M–X–H–F–R–W– motif, which serves as the pharmacophore for receptor binding (8, 10). The –K–K–R–R–P– motif within adrenocorticotropic hormone (ACTH) is considered the second pharmacophore and is required for successful activation of adrenocorticotropic hormone receptor (26).

Adrenocorticotropic hormone (ACTH), discovered in 1933 (1), is the primary regulator of aldosterone and corticosterone/cortisol production in mammalian adrenal glands (2–5). It is secreted into the circulating blood stream by corticotropic cells in the anterior pituitary (6) in response to short- and/or long-term stress (7). Due to its chemical structure, biological activity, and origin, ACTH is classified as a member of the peptide hormone group named melanocortins (MCs), which in addition to ACTH also comprises α-, β-, γ- and δ-melanocyte-stimulating hormones (MSHs), of which the latter is only found within cartilaginous fish (8). All MCs are formed through tissue- and site-specific proteolysis of the propeptide proopiomelanocortin (POMC; Figure 1) (7, 9), and all share the –M–X–H–F–R–W– consensus sequence (Figure 2), which is the main determinant of their biological activity (8, 10). Experimental data suggest that this core sequence adopts a β-turn secondary structure in most MCs that is generally essential for ligand binding (11). The exception to this rule is γ-MSH, but since it is not directly relevant to the main subject of this article, the reasons underlying this difference are not discussed [for details on molecular mechanisms underlying the action of this peptide see Ref. (12)]. α-MSH and ACTH are overlapping peptides since the aa sequence of the former is identical to the first 13 aa of the latter, because the former is the result of proteolytic cleavage of the latter (Figure 1). Following their discovery and aa sequence determination (1, 13–19), there was an ongoing discussion about the melanotropic activity of ACTH and the possible adrenocorticotropic activity of α-MSH (13, 20). However, the latter of these was soon dismissed following overwhelming evidence from *ex vivo* assays that suggested ACTH-derived peptides, which lack the basic –K–K–R–R– motif (Figure 2), have minimal effect on steroid production [(21, 22); reviewed in Ref. (23–25)]. Detailed mutational research performed a few decades later extended this motif by including a C-terminal proline, since the replacement of this residue with alanine or tryptophan significantly decreased the potency of the peptide (26). A similar alanine scanning mutagenesis experimental approach was also used to investigate the properties of the ACTH region, which is located between the –M–X–H–F–R–W– and –K–K–R–R–P– motifs (Figure 2). The results demonstrated that although replacement of one or two residues had no effect on receptor activation, substitution of all five residues (–G–K–P–V–G–) resulted in dramatic decrease in response sensitivity. Thus, it was concluded that the secondary structure of this motif is of paramount importance because it properly orients the other two motifs in relation to each other so that they can properly fit into the receptor-binding pocket(s) (27). This highly condensed introduction to ACTH, the ligand of ACTHR that is the main subject of this review, barely skims the enormous amount of knowledge acquired for mammalian and other lineages (6, 8, 10, 28–37). However, although having a detailed understanding of the functional properties of the ligand is very import because the interaction of both elements results in the physiological effect, reviewing the ligand in detail is beyond the scope of this article. We believe the core information provided here will be sufficient for interpretation of the research on the molecular mechanisms underlying the action of the adrenocorticotropic hormone receptor (ACTHR).

Although the main subunit of the receptor was discovered in 1992 (38) and the functional receptor was obtained in 2005 (39), research on the structure and function of the mammalian ACTHR dates back as far as 1954, when the aa sequence of ACTH was determined (14–18), or perhaps even to 1933, when the hormone itself was discovered (1). Although to some readers this statement might seem exaggerated, *ex vivo* research on the physiological effects of ACTH was performed on tissue samples containing endogenously expressed ACTHR from these dates. Thus, a large amount of knowledge was accumulated well before the discovery of the receptor itself, and some of these early studies revealed that the receptor was also present in murine fat cells (40–42) and in addition to increasing corticosteroid production (1, 43), activation of the ACTHR is accompanied by the activation of phosphorylases (44, 45), an increase in cyclic adenosine 3′,5′-monophosphate (cAMP) concentration (46–50), and subsequently desensitization (51). Research performed in the 1980s also revealed that stimulation with ACTH increases the number of ACTH-binding sites at adrenal cell membranes (52) and promotes calcium influx (53–56). The information gained during these studies was extensive, and it provided researchers with clues on the nature of the ACTHR and accelerated research in related areas.

At the beginning of the 1990s, a significant number of G-protein coupled receptors (GPCRs) were already identified, all of which share the typical seven transmembrane (TM) domain core structure with the N-terminus located on the exterior of the cell. These receptors were shown to activate intracellular signaling pathways through coupling to the α-subunit of heterotrimeric G-proteins (Gα) that comprise also β and γ subunits. At the time, it was already known that there are several types of G-proteins that induce distinct intracellular responses (57). The most notable of these from the perspective of melanocortin receptors (MCRs) and the ACTHR were those coupling to the Gα_s_ subunit that activates adenylyl cyclase, which in turn increases the intracellular concentration of cAMP. This knowledge was consistent with the observed ACTH-induced intracellular responses, which indicated that MCRs are members of this structurally conserved GPCR family.

The coding sequences of receptors that specifically bind ACTH were first reported in 1992 by two research groups, one led by Cone, and the other led by Wikberg (38, 58). Although both groups employed similar methodology (degenerate primer PCR followed by northern blot screening of melanoma cDNA libraries), the first group identified two receptor-encoding genes, and the second group identified one such gene. This discrepancy could partially be explained by the source of the PCR template, since Cone’s group used cDNAs from melanoma cells, while Wikberg’s group used genomic DNA. Subsequent pharmacological characterization of receptors and post-publication sequence comparison revealed that both groups discovered the same MCR, which is highly expressed within melanocytes and specifically binds all MCs (ACTH, α-, β- and γ-MSH), and was thus designated the MSH receptor (MSHR) (38, 58). In addition to MC1R, Cone’s group also discovered a receptor sharing significant aa sequence identity with MSHR (~39%), yet it proved to be difficult to characterize *in vitro*. Functional expression of this protein was achieved only in an endogenous MCR-expressing Cloudman S91 melanoma cell line, and unlike MSHR, this receptor was found to be expressed in adrenal tissue and activated only by ACTH and was hence named the ACTHR (38). In the following 2 years, three additional MC-binding receptors were uncovered—two by Yamada’s group (59, 60) and one by both the Wikberg’s and Yamada’s groups (61, 62). Due to this simultaneous discovery, there was some ambiguity in the nomenclature of these novel MCRs, but the scientific community soon came to agreement and named them using Yamada’s nomenclature as the MC type 3 receptor (MC3R), MC4R, and MC5R. Evaluation of the pharmacological properties of these novel receptors revealed that, like previously characterized homologs, they all coupled with Gα_s_ [it was later discovered that the exception to this rule is MC3R, which is also able to interact with the Gα_q/11_ subunit (63)]. However, from a ligand selectivity and *in vitro* expression perspective, they are more akin to MSHR since they are activated by all four MCs and successfully expressed in non-melanoma cells. Since these new receptors also shared significant aa sequence identity with previously discovered homologs (all MCRs share 39–61% sequence identity), their nomenclature was reviewed and, as a result, MSHR became synonymous with MC1R, and ACTHR with MC2R. To avoid any further confusion, in this article, we use ACTHR throughout, since this name better describes the unique functional properties, while the HGNC approved name of MC2R is a better descriptor of its evolutionary origin.

Considered together, the results on MCR expression and ligand selectivity highlighted the unique nature of each receptor because all displayed distinct pharmacological and tissue distribution profiles (Table 1). From this perspective, MC3R and ACTHR are the most specialized MCRs, because MC3R is the only receptor in this family that effectively binds γ-MSH and, as mentioned above, ACTHR specifically binds only ACTH (64). The molecular mechanisms underlying the ability of MC3R to effectively bind γ-MSH were soon uncovered (12, 65) following the expression of this receptor in a variety of mammalian cell lines. However, the mechanisms underlying the ACTHR ligand selectivity remained concealed for more than a decade due to the unusual expression selectivity of this receptor.

**Table 1:** **Ligand selectivity and expression profiles of melanocortin receptors**.

Receptor	Potency of ligands	Site of expression
MC1R	α-MSH = ACTH > β-MSH > γ-MSH	Melanocytes

ACTHR	ACTH	Adrenal cortex, adipocytes

MC3R	α-MSH = β-MSH = γ-MSH = ACTH	Hypothalamus, limbic system, placenta, digestive tract

MC4R	α-MSH = ACTH > β-MSH > γ-MSH	Hypothalamus, limbic system, cerebrum, brain stem

MC5R	α-MSH > ACTH > β-MSH > γ-MSH	Muscles, liver, spleen, lungs, brain, adipocytes

Although the period following the determination of the DNA sequence encoding the ACTHR was marked by very few molecular studies, this knowledge did facilitate functional and genetic research, which confirmed expression in the *zona reticularis* and *zona fasciculata* of the adrenal cortex, where its activation upregulates its own mRNA production (66). ACTHR mRNA was also found to be present in murine adipocytes (67–69) [where it affects lipolysis (67, 70) as well as leptin (71) and interleukin 6 production (69)], skin (72–74), pituitary (75), rat sympathetic ganglia (76), fetal and neonatal mouse testis (77, 78), human endometrium (79), human erythroblasts (80), and human osteoblasts (81, 82). The concept of receptor desensitization was not forgotten and was investigated in detail, and findings were somewhat contentious, but all agreed that receptor phosphorylation by various kinases (GPCR kinase and protein kinases A and C) is essential for desensitization and subsequent internalization *via* clathrin-coated pits (83–87).

There are several disorders associated with aberrant ACTH action. Familial glucocorticoid deficiency (FDG), which is also known as familial glucocorticoid insufficiency, hereditary adrenocortical unresponsiveness to ACTH, and familial Addison’s disease, is a rare, early onset, autosomal recessive disorder characterized by low or undetectable plasma cortisol levels, normal mineralocorticoid levels, excess plasma ACTH, and hypoplasia of adrenal cortex *zona fasciculata* and *zona reticularis*. Typical physical symptoms include frequent hypoglycemia and/or infective episodes accompanied by excessive skin pigmentation. This disease was described in detail for the first time by Shepard in 1959 (88), and other studies soon followed (89–96). All reports highlighted an unusual resistance to ACTH in patients and a positive response to glucocorticosteroid treatment. Nevertheless, until 1993, when the first mutations within *ACTHR* coding sequence were discovered (97), the genetic cause of this disorder remained unknown. From this pivotal moment, the number of reports involving sequencing of *ACTHR*, which is located on the small arm of chromosome 18 (18p11.21-pter) (98), increased significantly (99–101) and facilitated accumulation of some structural information that is reviewed in a separate chapter. However, although this information did provide some clear links between genetic cause and physiological effect, surprisingly, mutations within *ACTHR* were the cause of FGD in only 25% of cases, indicating additional ACTHR-related factors. In the aftermath of these initial genetic discoveries, the disorder caused by a faulty *ACTHR* gene was designated as FDG type I, while disorders of unknown cause were designated FDG type II (100, 102–104).

As the incoming information from genetic studies was in a good agreement with the difficulties in expressing ACTHR, interest remained intense and molecular research continued. The first breakthrough was achieved in 1995 when Schimmer and colleagues revealed that ACTH-resistant Y6 and OS3 cell lines derived a decade earlier from the Y1 adrenal cell line (105) completely failed to express ACTHR (106). This provided for the first time a platform for sensitive and unbiased ACTHR characterization (107–109). An important study using one of these cell lines was performed by Noon and colleagues who expressed an ACTHR-green fluorescent protein (GFP) fusion protein and observed that the receptor is retained within the endoplasmic reticulum (ER) of CHO cells, but successfully reached the cell membrane in Y6 cells. Thus, they concluded that adrenal cells produce some kind of ACTHR accessory factor that overcomes receptor trafficking arrest (110).

The discovery of this accessory factor was reported in 2005 by Metherell and colleagues (39) who performed SNP array genotyping on an FGD type 2 family to map the causal mutation to chromosome 21 locus 21q22.1, which spanned 30 known or predicted genes at the time. Subsequent *in silico* and mRNA expression analyses revealed that one of these genes, a predicted small single TM domain protein encoding a six exon gene named “chromosome 21 open reading frame 61 (C21orf61),” was expressed in adrenals and carried a mutation within the third intron donor splice site. *In vitro* co-expression of this protein with ACTHR-GFP in CHO and SK-N-SH cells confirmed that the receptor successfully reached the cell membrane and was functionally active. Based on these results, the newly identified protein was renamed the melanocortin receptor accessory protein (MRAP). Following the confirmation of its functional purpose, Matherell and colleagues performed an in-depth analysis of the gene and its expression profile. They discovered two isoforms of MRAP resulting from alternative splicing: MRAP-α and MRAP-β. The 172 aa MRAP-α is the product of the first five exons, while the 102 aa MRAP-β is formed from the third, fourth, and sixth exons. Since the “Start” codon is located within the third exon, both isoforms have identical N-termini and TM domains, but distinct C-termini. Analysis of protein expression patterns revealed that both proteins are differentially expressed in various tissues. Simultaneous expression was observed in the adrenals, testis, breast, ovary, adipocytes, skin, and jejunum. MRAP-α is also expressed in the thyroid, lymph nodes, ileum, liver, stomach, and pituitary, while MRAP-β is only expressed in the brain (39). It was later revealed that mutations in *MRAP* account for another 20–25% of FGD cases, thus it was established that there are additional causal factors for this disease that distinguish FGD type 2 and FGD type 3, the later referring to cases of unknown genetic cause (111).

As would be expected, the discovery of MRAP stimulated research aimed at uncovering the molecular mechanisms of ACTHR action. However, it also initiated an additional line of research aimed at investigating MRAP itself. These studies demonstrated that ACTHR is differentially affected by MRAP isoforms, with MRAP-α providing higher sensitivity to ACTH, whereas higher cAMP response and membrane trafficking occur during co-expression with MRAP-β (112). Detailed investigations on the actions of the accessory proteins revealed a unique feature; in the cell, MRAP is present as a highly stable, SDS-resistant, antiparallel homodimer in which the C-terminus of one monomer is on the cytoplasmic side of the cell membrane, while the C-terminus of the other is on the extracellular side of the cell membrane (113, 114). A few years later, the same dimer topology was also observed in another membrane protein sharing 39% aa sequence identity with MRAP that was also able to facilitate ACTHR trafficking to the cell membrane, and this evolutionary related protein was therefore named MRAP2 (115). There was, however, one major difference between MRAP1 (the name was accordingly updated) and MRAP2; when co-expressed with MRAP2, ACTHR was located on the surface of the cell, but stimulation with ACTH induced the cAMP response with 1000-fold lower potency than when co-expressed with MRAP1 (116). This observation indicated that MRAP1 is more than a mere “deliverer” and is instead an essential functional component of ACTHR (115). Indeed, it was soon discovered that the N-terminal region comprising residues 18–21 (–L–D–Y–L–) is essential for ACTHR ligand recognition (117). Its substitution with alanine residues resulted in an MRAP2-like protein that promoted only the trafficking of receptors to the plasma membrane. Alignment of both MRAPs revealed that this is the exact region missing from the MRAP2 sequence, and its insertion using site-directed mutagenesis allowed MRAP2 to form a functionally active ACTHR (117, 118). Further mutagenesis of various MRAP1 regions also revealed that the N-terminal side of TM region, formed from residues 31–37 (–L–K–A–N–K–H–S–), is required for MRAP1 to form an antiparallel dimer, since its removal resulted in the formation of a protein with the N-terminus always oriented on the extracellular side of the cell membrane (117). Additional aa replacement experiments showed that the TM domain of MRAP1 provides specific coupling to ACTHR, although the nature of this interaction remains unclear (117, 118). It should be noted that this review only considers MRAP1 in relation to ACTHR, but the functions of this protein extend beyond formation of functionally active ACTHR (115, 119–123).

**Figure 3:**
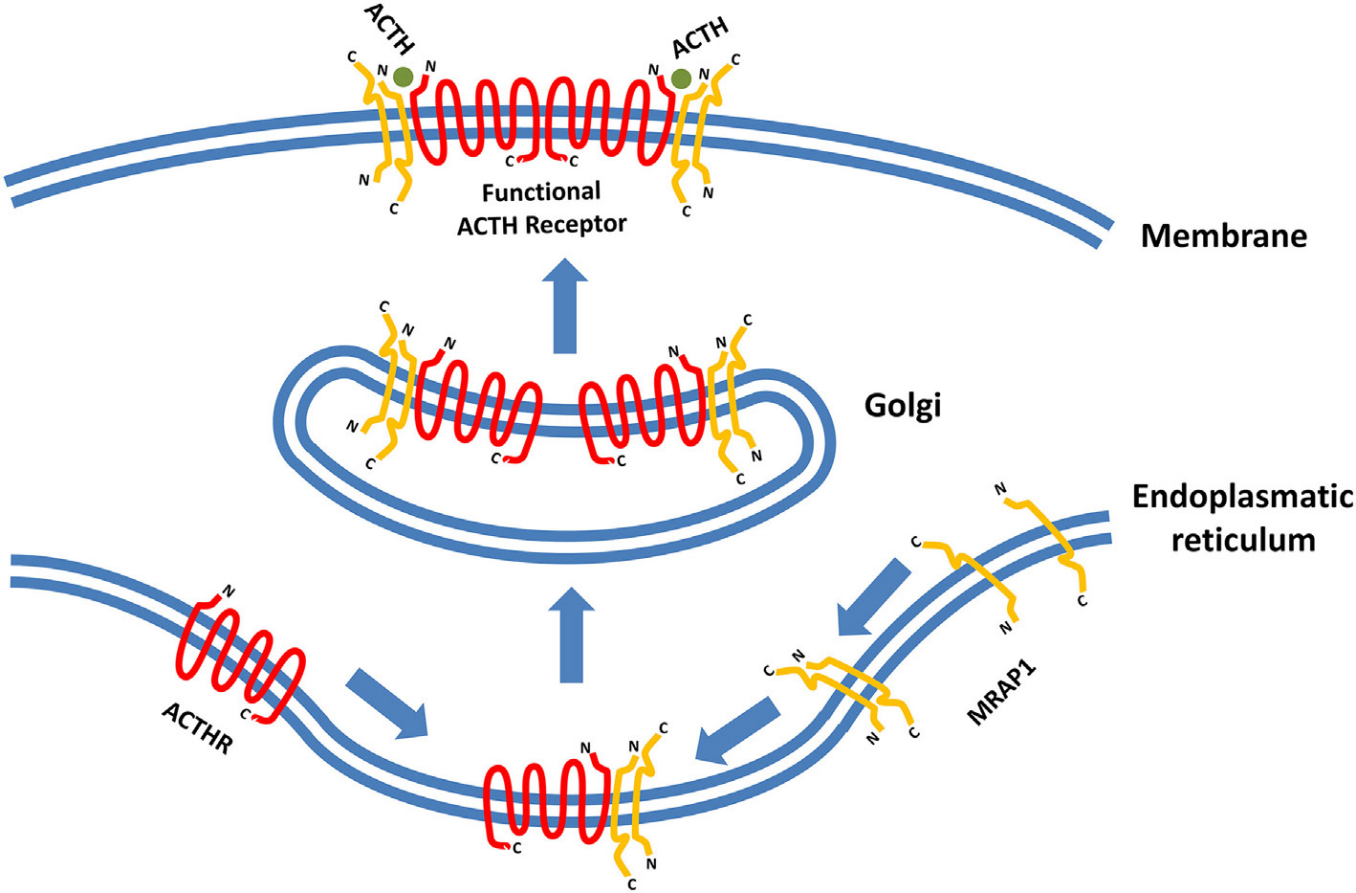
**Formation of functionally active adrenocorticotropic hormone receptor (ACTHR)**. The functional form of ACTHR is a hetero-hexameric structure formed from two molecules of ACTHR and four molecules of MRAP1. The antiparallel dimer of MRAP1 is formed within the endoplasmic reticulum, where it couples to one molecule of ACTHR. Afterward, this complex is transported to the Golgi where it “dimerizes,” thus forming the hetero-hexameric functional ACTHR (128). Symbols representing ACTHR and MRAP1 were selected to highlight the protein secondary structure. The depicted intermolecular interactions are therefore general and do not reflect the actual situation in detail.

The latest development in the field of ACTHR research regards the dimerization of the receptor itself. It has been known for a long time that various GPCRs form homo- and heterodimers and that dimerization is essential for the formation of functionally active receptors and implementation of their physiological effects (124–126). Therefore, the discovery of MCR homo- and heterodimerization was not surprising (127). However, initial difficulties with expression prevented such experiments on ACTHR, but this changed in 2011 when Cooray and colleagues performed bioluminescence resonance energy transfer analysis on functionally active ACTHR (the ACTHR and MRAP1 complex) which revealed that, just like other GPCRs, ACTHR formed dimers. Furthermore, only when each ACTHR subunit was accompanied by an MRAP1 dimer was a functionally active ACTHR formed, hence the realization that a hetero-hexameric structure is required for full activity (Figure 3) (128).

Finally, we would like to highlight the important contribution made by *ACTHR* knock-out mice. Phenotypic data from these animals were in a very good agreement with observations by medical practitioners on the effects of ACTHR deficiencies in humans. *ACTHR* knock-out mice therefore provided an experimental system for investigating the complexity of ACTHR action in mammals (129–134), but these studies are too voluminous and are beyond the immediate scope of this article.

## MCs and MCRs

More targeted research aimed at understanding the molecular mechanisms that underlie the action of MCRs was undertaken soon after their discovery. The majority of these studies focused on MC1R or MC4R, as they were either considered the best representatives, or the pharmacologically most relevant MCRs. Some research was also carried out on MC3R and MC5R, but due to expression difficulties, targeted studies on ACTHR were not performed until after 2000. Nevertheless, despite being highly specific, ACTHR is a member of the MCRs family and thus general MCR ligand-binding mechanisms are relevant and likely to apply.

Among the first to explore these mechanisms through active intervention were Frandberg and colleagues who performed PCR-based site-directed mutagenesis, a novel method at that time, to substitute selected aa residues with alanine to evaluate the role of four human MC1R aa residues (D^117^, H^260^, F^179^, and H^209^) in ligand recognition and receptor activation. The selection criteria for these residues were the location within the TM domains, the presence of reactive groups, and sequence conservation between known MCRs. The employment of the last two criteria was somewhat traditional, since reactive groups are necessary for molecular interactions, whereas the degree of conservation indicates structural and/or functional importance of residue. However, the first criterion was unusual because known binding pockets for the majority of receptors were on their surface. MCRs were the smallest known GPCRs, and they bind relatively large peptide ligands, thus it was predicted that their binding pocket would be located between the helices. The results of these experiments revealed that two residues of the four (D^117^ and H^260^) were essential for binding of α-, β- and γ-MSH peptides, while their substitution had no effect on coupling with artificial ligand NDP-MSH ([Nle^4^,d-Phe^7^]-α-MSH). This demonstrated not only the importance of these residues but also the principle that different ligands may interact with different regions of the binding pocket (135).

**Figure 4:**
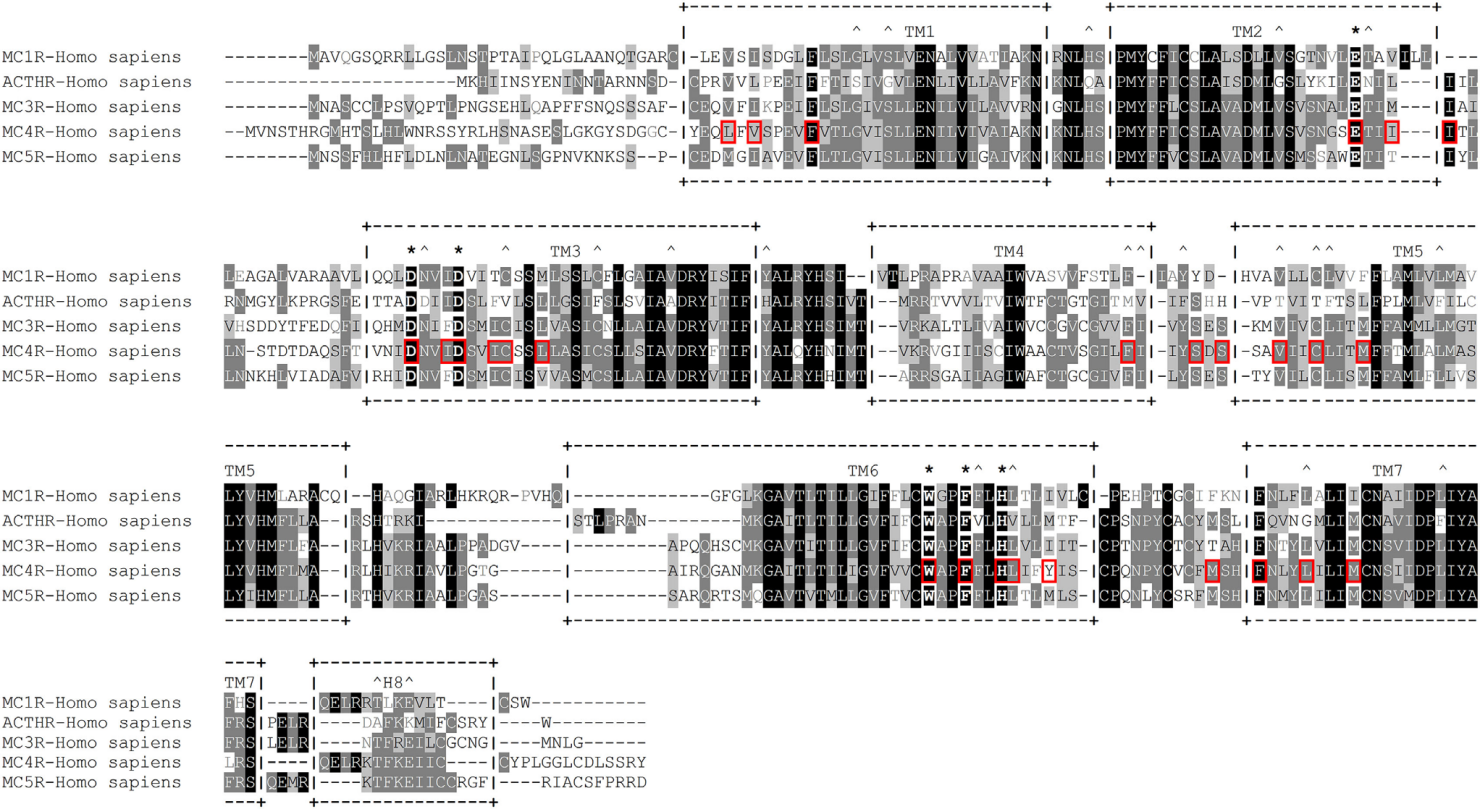
**Amino acid sequence alignment of human melanocortin receptors (MCRs)**. +--+ and vertical dashes mark transmembrane domain boundaries, acquired from GPCRdb (http://gpcrdb.org/). *Indicates residues that form the “backbone” of the MCR-binding pocket. ^^^Indicates residues that are unique to adrenocorticotropic hormone receptor (ACTHR). Red boxes indicate amino acid residues that, according to the model created by Pogozheva et al. (148) and Chai et al. (149), form the ligand-binding cavity of MC4R.

Researchers employed a more sophisticated approach in one subsequent study; instead of creating a large number of receptors by mutating conserved residues, they generated a three-dimensional molecular model of human MC1R based on the cryo-electron microscopy structure of bacteriorhodopsin and the electron density footprint of bovine rhodopsin and docked several natural and synthetic ligands. The results suggested the cavity of MC1R consists of two distinct acidic- and aromatic-binding pockets. According to this model, the acidic pocket is formed from E^94^ (TM2), D^117^ (TM3), and D^121^ (TM3), while the aromatic pocket is formed from F^175^ (TM4), F^179^ (TM4), F^195^ (TM5), F^196^ (TM5), F^257^ (TM6), Y^182^ (TM4), and/or Y^183^ (TM4). Being acidic, the first pocket interacts with basic R residues and to some extent H residues in the MC consensus motif –M–X–H–F–R–W–, while the second pocket interacts with aromatic F and W residues of the same motif (136). In the following years, this research group rigorously tested this model with various point mutations, different native and synthetic ligands, and even through conversion to MC4R. Their results confirmed that residues in the predicted acidic pocket were of paramount importance for ligand binding and do indeed interact predominantly with R residue from the –M–X–H–F–R–W– motif. Data on the aromatic-binding pocket were harder to interpret because considerable alterations in ligand binding were achieved only when multiple mutations were introduced at the same time, suggesting each individual residue plays a small part, and the loss of one may be compensated by the others. However, this conclusion was not supported by an acidic pocket eradication experiment in which all three pocket-forming residues were replaced with alanines. The resulting receptor was functionally inactive yet able to bind α-MSH with modest affinity, indicating other potentially more important ligand-binding elements within the aromatic pocket. Thus, the repertoire of mutated aromatic residues was extended, and W^254^ (TM6), Phe^257^ (TM6), and H^260^ (TM6) were recognized as crucial elements of this ligand-binding pocket (137–139). In the following years, this type of research was performed on all five MCRs, which highlighted the uniqueness of the individual receptor-binding pockets by identifying the aa residues responsible for differential ligand recognition. However, all studies agreed that the six previously mentioned aa residues (Figure 4) form the “backbone” of the MCR-binding pocket, while the residues surrounding these act as binding pocket “modifiers” [(65, 140–143); reviewed in Ref. (144–147)].

**Figure 5:**
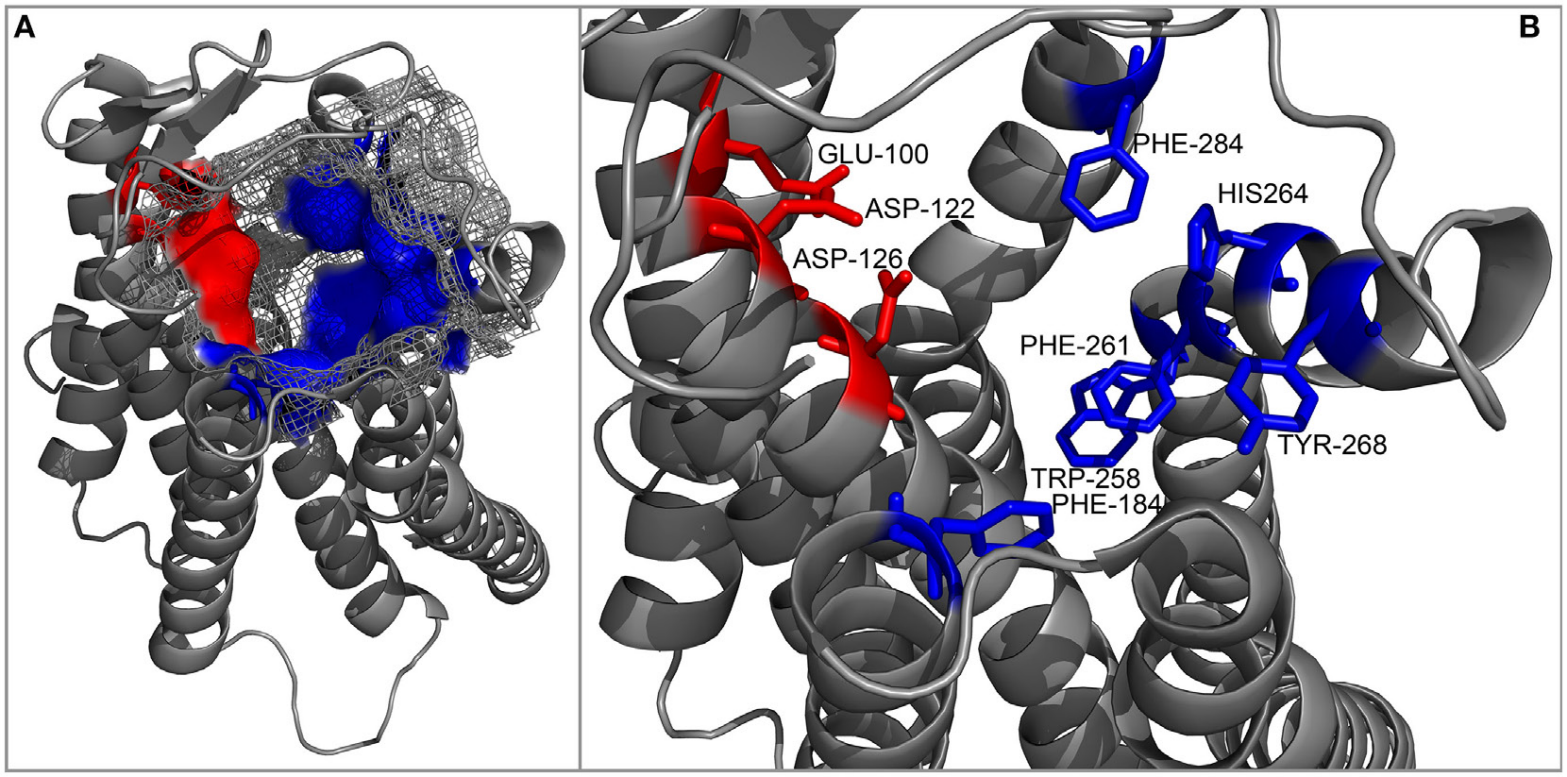
**Cartoon representation of the backbone of the MC4R model created by Pogozheva et al. (148) and Chai et al. (149)**. **(A)** View from the side and top on the surface of the ligand-binding cavity that contains several acidic (red) and multiple aromatic residues (blue) located in groups at opposite sides of the cavity and forming acidic- and aromatic-binding pockets, respectively. **(B)** Close-up view of the binding cavity without the surface layer.

An interesting attempt to redesign the three-dimensional model of MC4R was undertaken by Pogozheva et al. (149) and Chai et al. (150). Unlike previous groups, these researchers employed data from functional analysis of receptors with point mutations and also used the crystal structure of bovine rhodopsin which had been recently determined (150). In addition, to test the validity of their model, they also performed docking of various native and artificial ligands. According to their model, the binding pocket of MC4R is a large elongated cavity formed by L^44^, V^46^, F^51^, E^100^, I^103^, I^104^, D^122^, I^125^, D^126^, I^129^, C^130^, L^133^, F^184^, S^188^, S^190^, V^193^, C^196^, M^200^, W^258^, F^261^, H^264^, L^265^, Y^268^, M^281^, F^284^, L^288^, and M^292^. This cavity contains several acidic and multiple aromatic residues located in groups on opposing sides thus forming the predicted-binding pockets with “backbone” MC-binding residues (Figures 4 and 5). Although hypothetical, this model clearly demonstrates that a significant amount of space is required to incorporate the relatively large β-turn structure of MCs (148), and a similar situation would be expected with all MCRs (links to models are available at http://www.uniprot.org/uniprot/P32245).

In the following years, a number of groups generated 3D models of various MCRs, and each explored MCR-specific themes, whether mutation-induced structural changes or differences in ligand recognition. However, the majority of these models are unavailable for download, and we were therefore unable to assess their intramolecular and intermolecular interactions in detail. For this reason, we used the model generated by Pogozheva et al. (148) and Chai et al. (149) as a reference in our following analysis.

## Naturally Occurring Mutations

Historically, the first source that provided information on the molecular mechanisms underlining the action of ACTHR were genetic studies on FGD—a rare autosomal recessive disorder that, as mentioned in Section “Introduction,” can be caused by defects in the *ACTHR* gene. From a genetic standpoint, all these alterations can be subdivided in three groups: mutations within regulatory sequences, mutations that result in severely truncated proteins (frameshift mutations or mutations that introduce STOP codons), and mutations that alter a single aa residue. Since the first two groups, from perspective of this article, are uninformative, they are not reviewed in detail [for reports on this subject see Ref. (107, 151–160)]. However, the third group has provided the scientific community with some valuable knowledge.

**Figure 6:**
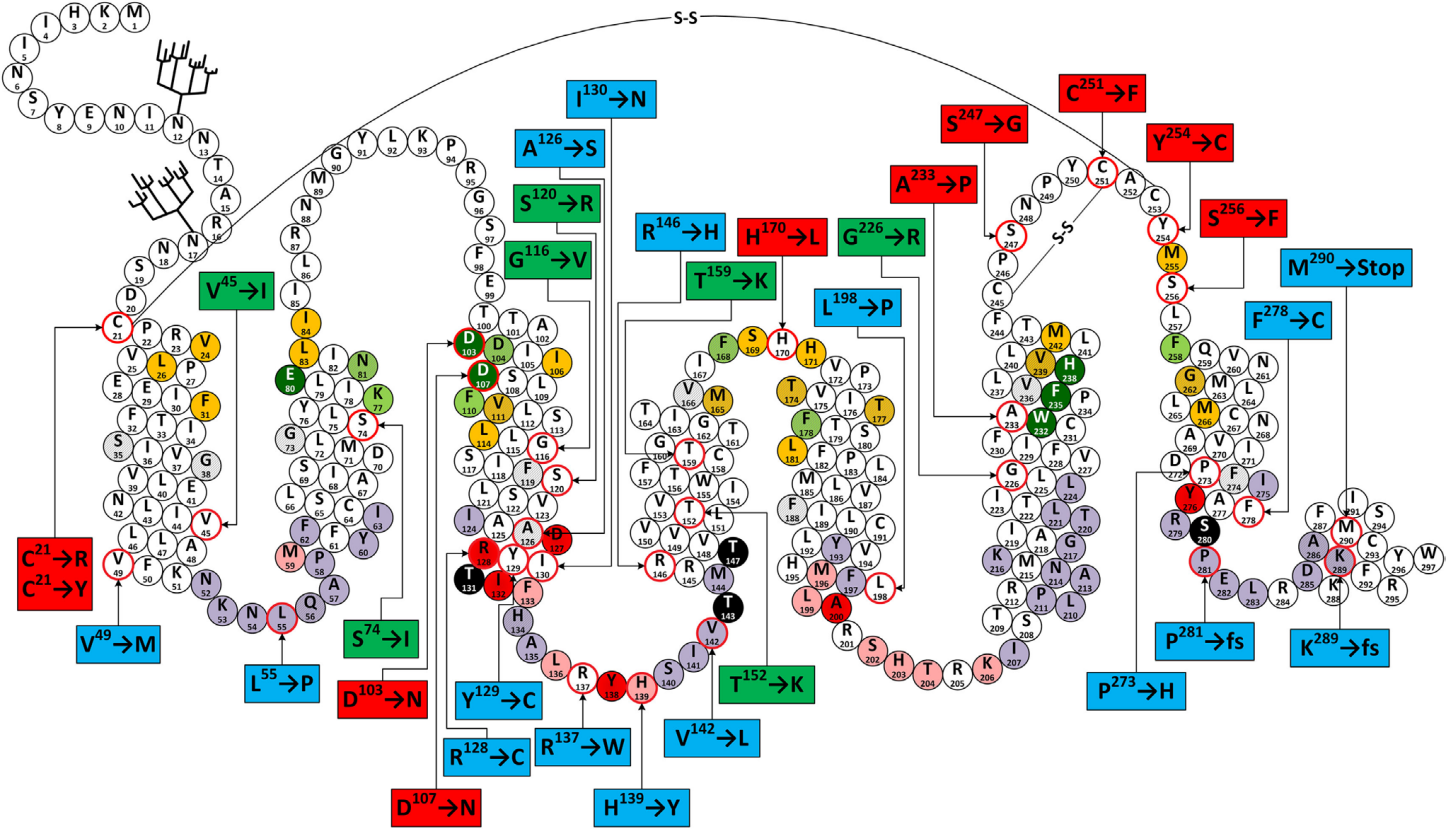
**Snake-like plot of adrenocorticotropic hormone receptor (ACTHR)**. Forked structures represent glycosylation. –S–S– represents disulfide bonds. Circles with a red outline represent naturally occurring missense mutations (labels enclose information on the resulting residue replacement). Red labels indicate residues located on the extracellular part of the receptor. Green labels indicate residues located within the central part of transmembrane domains. Blue labels indicate residues located within the intracellular part of receptor. Dark green circles represent residues that form the backbone of the melanocortin receptor (MCR)-binding pocket. Orange circles represent residues equivalent to those forming the ligand-binding cavity of MC4R based on the model created by Pogozheva et al. (148) and Chai et al. (149). Light green circles represent residues that Yang et al. (146) identified as important for ligand recognition. Circles shaded in red, pink, and light purple represent residues equivalent to those that, according to the ADRB2 model (163), directly interact with Gα, are involved in Gα coupling-related intramolecular interactions, and that form the surface of the Gα-binding cavity, respectively. Circles shaded in black represent S/T residues that are crucial for ACTHR expression and functional regulation. Circles with angled cross-hatching represent residues that are unique to ACTHR (yet conserved in other MCRs).

The first report on a missense mutation within FGD patients was published by Clark and colleagues soon after the discovery of the ACTHR coding sequence (97). The mutation was located within the extracellular part of the TM2 domain and resulted in substitution of S^74^ with I (Figure 6). Subsequent functional characterization of the mutated receptor confirmed that its ability to induce cAMP (107, 161, 162), bind ACTH (107), and even reach the cellular surface (despite being able to couple to MRAP1) (156) was severely reduced in comparison to wild-type (WT) ACTHR. As expected, the discovery of this mutation was soon followed by others, and at present 35 different coding sequence alleles have been associated with adrenocortical disorders (detailed information on these mutations is summarized in Table 2; Figure 6).

**Table 2:** **Amino acid residue replacements of naturally occurring *ACTHR* gene missense mutations and their effects on receptor function**.

Mutation	Binding	Cyclic adenosine 3′,5′-monophosphate (cAMP)	Basal cAMP activity	Membrane trafficking	Reference
_EC_C^21^ → R	↓		↔		(164, 165)

_EC_C^21^ → Y					(166)

_TM_V^45^ → I	↔	↔			(159)

_IC_V^49^ → M					(153)

_IC_L^55^ → P				↓	(156)

_TM_S^74^ → I	↓	↓		↓	(97, 107, 108, 156–158, 161, 162)

_EC_D^103^ → N	↓	↓	↑	↔	(107, 156, 167–170)

_EC_D^107^ → N	↓	↓		↔	(156, 159, 171–173)

_TM_G^116^ → V		↓		↓	(156, 174)

_TM_S^120^ → R				↓	(151, 156)

_IC_A^126^ → S		↓			(160)

_IC_R^128^ → C	↓	↓		↔	(107, 156, 158)

_IC_Y^129^ → C		↓		↓	(156, 175)

_IC_I^130^ → N				↓	(156, 176)

_IC_R^137^ → W		↓		↓	(108, 156)

_IC_H^139^ → Y				↓	(156, 176)

_IC_V^142^ → L		↓			(167–169)

_IC_R^146^ → H	↓	↓	↑	↓	(107, 156–158, 166, 177)

_TM_T^152^ → K				↓	(156, 178)

_TM_T^159^ → K	↓	↓	↑	↓	(107, 156, 179)

_EC_H^170^ → L		↓		↔	(156)

_IC_L^198^ → P				↓	(156, 176)

_TM_G^226^ → R				↓	(156)

_EC_A^233^ → P		↓		↓	(156, 168, 169)

_EC_S^247^ → G	↓		↔		(164, 165)

_EC_C^251^ → F		↓		↓	(156, 159, 171, 172)

_EC_Y^254^ → C		↓		↓	(108, 156, 180, 181)

_EC_S^256^ → F				↓	(156)

_IC_P^273^ → H		↓		↓	(156, 162)

_IC_F^278^ → C	↔	↔	↑	↔	(109, 175)

_IC_P^281^ → fs					(173)

_IC_K^289^ → fs				↓	(182)

_IC_M^290^ → Stop				↓	(182)

_EC_C^21^ → R + _EC_S^247^ → G			↑		(164)

_IC_Y^129^ → C + _IC_F^278^ → C		↓		↓	(175)

*EC, located within the extracellular part; IC, located within the intracellular part; TM, located within the central part of the TM domain; fs, frameshift; ↔, functional properties were unaffected; ↑, functional properties were improved; ↓, functional properties were hampered; empty field, no data available*.

From a functional point of view, these mutations can be divided into three groups: (1) those located within the intracellular part of the receptor (Table 2; blue labels in Figure 6) that are most likely to affect G-protein interactions; (2) those located on the extracellular part of the receptor (Table 2; red labels in Figure 6) that are most likely to affect receptor–ligand interactions; (3) those located within the central regions of TM domains (Table 2; green labels in Figure 6) that are most likely to affect the overall conformation of the receptor. Despite the fact that this division is quite general, it is in good agreement with data from modeling of the MCR-binding pocket (148) and also the crystal structure of the β_2_ adrenergic receptor–G_s_ protein complex (163).

The majority of extracellular mutations altered either the previously mentioned “backbone” MC-binding residues (dark green in Figure 6), as is the case for D^103^ and D^107^, or residues that are adjacent to other MC ligand cavity-forming residues (orange in Figure 6). According to the model, MC4R-S^259^ (ACTHR-A^233^) and the conserved MC4R-P^260^ (ACTHR-P^234^) are located between aromatic-binding pocket-forming residues MC4R-W^258^ (ACTHR-W^232^) and MC4R-F^261^ (ACTHR-F^235^) (Figures 4 and 6). The presence of conserved P within the TM domain is rather unusual, since due to its chemical structure it is unable to participate in the formation of an α-helix, and when present it introduces a “kink”-like structure that is often employed for the proper positioning of surrounding reactive aa residues. Indeed, this appears to be exactly the case for MC4R-P^260^ (ACTHR-P^234^), and it is logical to presume that additional proline located adjacent to this conserved one, as is the case of ACTHR-A^233^ → P, would not only disrupt the binding pocket but also alter the overall conformation of the receptor to the extent that it is recognized as misfolded and retained within intracellular compartments and ultimately degraded.

According to both the model and other experimental data, there are two structure-stabilizing disulfide bonds within the extracellular region of MCRs (148, 183, 184). It is presumed that in the case of ACTHR these are formed between C^245^ and C^251^, and between C^21^ and C^253^ (Figure 6). Both the MC4R model and predictions using the NetTurnP 1.0 server (185) indicate that the bond between this first pair of residues “locks” the conserved –P–Q–N–P–Y– motif of extracellular loop (EL) 3 into a β-turn-like structure, thus exposing its N and Y residues to the entrance of the binding pocket, and this also limits the physical distance between TM6 and TM7. Although data on the role of “locked” aa residues in MCR ligand recognition is ambiguous, the disruption of this disulfide bond (C^251^ → F) apparently prevents proper receptor folding and results in intracellular retention. From this perspective, it is hard to explain the negative effect of the S^247^ → G mutation, because according to model, the side chain of the residue homologous to ACTHR-S^247^ in MC4R is located on the exterior surface of the receptor and thus cannot be involved in ligand recognition. Perhaps the function of this β-turn-like structure is to serve as a physical barrier that restricts the movement of the ligand while it occupies the cavity, or as a physical support that keeps the cavity entrance open. Thus, replacement of S^274^ with most other residues would not have any effect, but replacement with the highly flexible G allows it to adopt an alternative conformation that does not provide the necessary physical support. The disulfide bond between C^21^ and C^253^ connects TM1 and TM7 domains and locks the TM domain bundle in a “closed” conformation, which reduces flexibility and the number of available conformations. This effect appears to be crucial for formation of a functionally active receptor, as the disruption of this bond by C^21^ → R or C^21^ → Y mutations resulted in receptors incapable of binding ACTH. One possible explanation is probably connected to fact that the surface of the MCR ligand-binding cavity is predominantly formed from the TM domains. Another mutation that falls within the disulfide bond category is Y^254^ → C, since it introduces an additional C residue within the EL3 region that could interfere with the formation of one or other of the previously mentioned disulfide bonds. Therefore, unsurprisingly, the Y^254^ → C mutant was intracellularly retained, similar to the C^251^ → F mutant.

The effects of the last two extracellular mutations (S^256^ → F and H^170^ → L) are harder to explain. Alignment of MCR coding sequences reveals that residue S^256^ is somewhat conserved (present in three out of five human MCRs), while the model suggests that the side chain of MC4R-S^282^ (ACTHR-S^256^) is oriented toward the external surface of the receptor and thus cannot directly interact with the ligand. However, it is located adjacent to MC4R-M^281^ (ACTHR-M^255^) that lines the binding cavity, and in close proximity to the disulfide bond forming MC4R-C^279^ (ACTHR-C^253^). Thus, replacement of the small, polar, and hydrophilic S with a bulky, non-polar, and hydrophobic F could affect these functionally important residues by misfolding the extracellular region, resulting in the retention of the receptor in the interior of the cell. The mechanisms of this effect may vary; for example, to minimize contact with water molecules, the side chain of F could reorient from the exterior surface to the internal cavity of the receptor, or as predicted by TMpred software (186), increase the length of TM7 by introducing an additional turn so that it is embedded within the lipid bilayer of the membrane.

Although difficult to explain, the H^170^ → L variant is perhaps the most interesting mutation of on the extracellular side of ACTHR because it is located within EL2, which is the shortest of the three loops. In most MCRs, this region is well conserved and formed from one acidic residue (E or D) and a number of hydrophilic and/or polar residues (Figure 4), but within ACTHR there are two basic H and one hydrophilic S residue. The conservation of acidic residues and the participation of both flanking residues in the formation of the binding cavity of MC4R are indicative of a functional purpose. Perhaps it works as “bait” for basic residues on the MC pharmacophore to recruit/direct ligand to the cavity entrance. Alternatively, it could interact with the basic –K–K–R–R–P– motif of ACTH, thus modulating its receptor activation ability. Determination of the true purpose clearly requires additional research. Nevertheless, such speculation is at least partially untrue in the case of ACTHR because the basic H^170^ is in the exact position occupied by an acidic residue in other MCRs, yet the loss of functional activity observed by Clark’s group (156) shows that it is still important. Peculiarly, the Clark group also observed that H^170^ → L, D^103^ → N, D^107^ → N, and R^128^ → C were the only 4 out of 22 functionally hampered receptors that were effectively transported to the cell membrane. Unifying the other three mutations was their location and functional purpose, since all are located deep within the cavities of ACTHR, and all participate in ligand–receptor or receptor–G-protein interactions. Thus, it can be presumed that these substitutions altered only the internal surface and not the overall structure of ACTHR. In light of this knowledge, it is tempting to speculate that H^170^ → L could have similar effects. However, being located on the edge of a binding cavity, this residue cannot participate in interactions with the MC consensus pharmacophore and must instead interact with either the second ACTH pharmacophore (–K–K–R–R–P–) or with MRAP1.

According to the MC4R model, mutations in the central part of the TM domains are located either on the external surface of ACTHR and thus in contact with the lipid bilayer (S^74^ → I T^152^ → K, T^159^ → K, and G^226^ → R) or buried within the tightly packed core of the TM bundle (S^120^ → R and G^116^ → V). Therefore, it was not surprising that all native residues were small and/or hydrophobic, and mutated residues were hydrophilic (S^120^ → R, T^152^ → K, T^159^ → K, and G^226^ → R) or significantly larger than native residues (S^74^ → I and G^116^ → V). The mechanisms underlying the changes in receptor activity for all these substitutions thus appear to be very simple. In the case of hydrophobic → hydrophilic alterations, the introduced residue would likely induce a rotational shift of the TM domain to minimize exposure to the hydrophobic environment, while for small → large alterations, the surrounding structure would have to shift to accommodate the larger residue. Either type of rearrangement would likely result in misfolding and/or altered functional cavities.

Analogous to mutations located on the extracellular side of receptor, those on the intracellular side likely affect receptor–G-protein interactions. The crystal structure of the β_2_ adrenergic receptor–Gs protein complex identified a number of receptor residues that are involved in interactions between both partners (163). Although the β_2_ adrenergic receptor (ADRB2) and ACTHR are relatively distantly related (~26% sequence identity), they both couple with the Gα_s_ subunit, thus the ACTHR surface that interacts with Gα should be similarly folded to that of ADRB2 (Figure 6). For this very reason, we based our subsequent analysis described in the next paragraphs on this receptor–ligand complex crystal structure (PDB ID: 3SN6).

Given that there are a significantly larger number of GPCR subtypes than G-protein subtypes, the correct folding of the intracellular parts of receptors is presumably rigorously monitored and misfolded molecules intracellularly retained and degraded. Indeed, this was observed by the Clark group (156) who characterized a number of intracellular mutations located within ACTHR regions that either directly interact with Gα_s_ (R^128^ → C) or are homologous to residues that form the surface of ADRB2 Gα_s_ coupling cavity (L^55^ → P, H^139^ → Y, V^142^ → L, P^281^ → fs, and K^289^ → fs). The effect of R^128^ → C can be easily explained because it is located within the highly conserved rhodopsin GPCR family –D–R–Y– motif, which is involved in signal transduction. The retention of L^55^ → P, H^139^ → Y, and V^142^ → L is most probably caused by relevant changes in the properties of the residues. Mutation to P often restricts the backbone of the polypeptide chain and prevents the adoption of its native conformation, while the replacement of more hydrophilic H and V to more hydrophobic Y and L generally causes local structural rearrangements because the introduced residues tend to get buried deeper within the protein or lipid bilayer to minimize contact with water molecules. In the case of V^142^, these local rearrangements and the increased size of the hydrophobic side chain could possibly affect phosphorylation of the adjacent T^143^ because the homologous position (T^157^) within MC1R is critical for receptor functional activity (86, 187, 188). Peculiar, however, were the P^281^ and K^289^ frameshifts and the M^290^ → Stop mutation affecting located within the small C-terminal intracellular α-helix (often referred as H8). Since neither structural data nor experimental results have illuminated the role of this region in signal transduction *via* G-protein binding, the retention caused by its loss seems interesting. Several research groups have undertaken efforts to uncover the purpose of this structural element, but results are ambiguous (189–191). Its primary role could be to properly orient the GPCR within the plasma membrane, so that it can be effectively accessed by both ligand and G-protein.

Results from mutations located outside the binding cavity are harder to interpret, yet some appear relatively straightforward. Substitution of Y^129^ → C, located within the previously mentioned –D–R–Y– motif, can be interpreted based on the ADRB2 structure, which suggests this particular residue is oriented toward the exterior of the molecule, and a role in signal transduction has been demonstrated in other MCRs (192). The A^126^ → S and I^130^ → N replacements involve changes from hydrophobic to hydrophilic residues. In both cases, the side chains of native residues are predicted to be located on the surface of the receptor and oriented toward the lipid bilayer; therefore, these replacements most probably induce some structural rearrangements so that the mutated residues have limited interaction with the hydrophobic environment of the membrane. The opposite is likely true of R^137^ → W, in which the hydrophilic R is replaced with the hydrophobic W. R^137^ (ADRB2-K^140^) is located between Y^138^ (ADRB2-Y^141^), and L^136^ (ADRB2-F^139^), and the first of these two residues interacts with D in the –D–R–Y– motif that orients the second residue toward the hydrophobic pocket of the G-protein. It appears the purpose of the basic R^137^ is to serve as a kind of a “lever” that keeps this otherwise hydrophobic region at the surface of the cell membrane. Its replacement would likely cause this region to adopt a different conformation and possibly “sink” deeper in to the membrane. As already mentioned, replacements involving P are often associated with large changes in the protein backbone, and this appears to be the case for P^273^ → H and L^198^ → P. P^273^ (ADRB2-P^323^) is one of the most highly conserved residues within rhodopsin GPCRs, where it introduces a kink in TM7 that allows the highly conserved Y^276^ (ADRB2-Y^326^) to interact with Gα_s_. Introduction of H at this position would likely disrupt the native conformation. The opposite is true of L^198^ which is located at the base of TM5 and followed by IL3, which plays a crucial role in receptor–G-protein interactions. Introduction of P and an associated α-helical kink would alter the orientation of the entire loop, resulting in a misfolded receptor with hampered signal transduction ability.

Of all the intracellular mutations, the effects of V^49^ → M and R^146^ → H are hardest to interpret, because in both cases the native residue is replaced with a residue that has similar properties. Nevertheless, closer examination involving analysis of the ADRB2 structure can shed light on the underlying mechanisms. Replacement of V^49^ → M involves only a change in size and the introduction of a sulfur atom. V^49^ (ADRB2-I^58^) is located on the surface at the base of TM1 and being hydrophobic, it does not protrude outwards but is instead oriented along the surface of the ACTHR molecule toward the TM2 domain and the receptor interior. V is a small residue compared with M, therefore the size difference could account for the observed effects. However, in ADRB2, the same position is occupied by I, which is also larger than V, yet this receptor has high activity, indicating that the introduction of a sulfur atom cannot be excluded. Interestingly, both sulfur-containing residues (C and M) and H are able to bind metal ions, and if several similar residues are present in a given location, they may simultaneously bind single metal ion to form a bridge-like structure. According to the crystal structure of ADRB2, the base of both TM1 and TM2 is located in close proximity, and there is an additional M^59^ at the base of the TM2 domain in ACTHR that may be sufficiently close to form a metal-binding site with the mutated M^49^ (V^49^ → M). Based on the structural similarity with ADRB2, this second M^59^ (ADRB2-T^68^) is likely located within the functionally important region that interacts with the previously mentioned Y^138^ (ADRB2-Y^141^) and D^127^ (ADRB2-D^130^) from the –D–R–Y– motif, hence the formation of this putative metal bridge-like structure could alter the ability of the receptor to activate the G-protein. Although this theory seems highly speculative, in our previous studies, we successfully introduced similar albeit artificial metal-binding sites into the structure of MC4R (147).

The R^146^ → H replacement involves residues that both are basic, suggesting the observed decrease in receptor’s ability to bind ligand, induce functional response and reach cellular surface is due to size differences, aromatic interactions, or the ability to bind metal ions rather than the loss of a reactive group. R^146^ (ADRB2-K^149^) is located on the surface of the receptor at the base of the TM4 domain. Being hydrophilic, this residue is oriented toward the surface of the molecule, thus structural alterations due to size differences are unlikely to affect receptor function. Coincidentally, as with the TM1 domain, the base of this domain is also located in close proximity to the TM2 domain. Therefore, the formation of a M^59^–H^146^ metal-binding pocket that alters the conformation of the intracellular region is also plausible. Additionally, within the TM2 domain, approximately one α-helical turn above M^59^ is located F^62^ (ADRB2-F^71^), which is conserved in rhodopsin GPCRs. The side chain of this residue is oriented toward the TM4 domain, suggesting that abnormal aromatic interactions could prohibit ACTHR from adopting its native conformation in a similar way to that proposed for the putative metal-binding pockets described above.

In addition to mutations that hamper receptor activity, the intracellular part of ACTHR includes two constitutive activity mutations F^278^ → C and the double mutation C^21^ → R + S^247^ → G. The first of these mutations was discovered in a patient with ACTH-independent Cushing’s syndrome, a disease typically caused by excess ACTH. Functional analysis revealed that this mutated receptor was effectively transported to the cell surface and displayed functional activity parameters that were comparable with WT ACTHR. The only difference was the elevated basal activity. Further research revealed that this effect was due to deficient receptor desensitization, and site-directed mutation of the phosphorylation site in the S^280^ → A variant resulted in a receptor with similar properties (109). The authors of this article concluded that the F^278^ → C mutation somehow masks the presence of the phosphorylation site. However, the mechanism of this masking is still unknown and hard to interpret. Some years later, a patient was identified that in addition to F^278^ → C also carried an inactivating Y^129^ → C mutation (as described in previous paragraphs). As a result, this double mutant receptor was intracellularly retained.

Although considered to be rare, this C^21^ → R + S^247^ → G double mutant was identified in another ACTH hypersensitivity patient (164). However, unlike in the previous patient, this mutant displayed only basal activity, and cAMP accumulation was not observed upon stimulation with ACTH. Expressed separately, both mutations generated functionally inactive receptors (as described in previous paragraphs), thus the authors speculated that within the double mutant these residues are located in close proximity and are required to maintain the receptor in an activated conformation.

Taken together, the knowledge gained from naturally occurring mutations is in good agreement with the findings of Pogozheva et al. (148), the MC4R-binding pocket model, and the crystal structure of the β_2_ adrenergic receptor–Gs protein complex of Rasmussen et al. (163). Thus, both models appear to be useful for evaluation of the effects caused by single residue mutations.

## Site-Directed Mutagenesis

Although analysis of naturally occurring mutations has provided us with a significant amount of functional data, the nature of this type of research can be somewhat random and unfocused. Thus, in order to pursue a broader knowledge on ACTHR specificity and the underlying molecular mechanisms, several research groups employed site-directed mutagenesis and subsequent functional characterization of mutated ACTHRs. Due to the difficulties to express functionally active ACTHR (193), majority of such studies were performed only after the characterization of Y6 and OS3 adrenal cell lines (106) and/or discovery of MRAP1 (39).

One of the first to perform systematic mutagenesis of ACTHR was Yang and colleagues who had already performed similar analyses on other MCRs (138, 139, 142). They introduced 16 point mutations within various ACTHR regions to assess their functional role in ligand binding and receptor activation. The repertoire of mutations covered both residues that were known to alter the activity of MC1-3R, and residues that, according to sequence alignment, were unique to ACTHR. The results of these analyses are summarized in Table 3 (146). In Section “Discussion,” the authors concluded that the general organization of the ACTHR ligand-binding cavity is similar to that of other MCRs, as it also consists of acidic- and aromatic-binding pockets formed by the same “backbone” residues (E^80^, D^103^, D^107^, F^235^, and H^238^). However, there are also significant differences. Compared with other MCRs, the role of D^103^ in ligand binding and receptor activation is significantly diminished, as its alteration caused only a 2.8-fold decrease in ACTH (1–24) affinity and a 7.3-fold decrease in potency. The role of this residue appears to be compensated by the adjacent D^104^ that is unique to ACTHR, since replacement of this residue had a greater effect (10.2-fold and 18.3-fold decrease, respectively). A number of aromatic residues unique to ACTHR were also tested, and their replacement with alanine had a significant (F^168^) or even tremendous (F^110^ and F^178^) effect on ligand binding and receptor activation. This led to speculation that the binding cavity of ACTHR could be “broader” than that of other MCRs and could accommodate additional ligand residues that would interact with some of the identified functionally relevant unique residues (146). However, work based on the MC4R-binding pocket model (148) partially counters this speculation, because homologous positions of these high effect aromatic residues (F^110^ and F^178^) were located in close proximity to aromatic residues of the MC consensus pharmacophore (F^235^ and W^232^, respectively), thus indicating their role in ligand binding and receptor activation. Nevertheless, this “broader” binding cavity remained possible because homologous position of the basic ACTHR-K^77^ within MC4R model resides within the densely packed and hydrophobic TM domain bundle region adjacent to the acidic D^103^. Being charged and larger than N (in MC1, MC3, and MC4R) or S (in MC5R), this residue could conceivably thread through this region toward the surface of the binding cavity and interact with D^103^ to alter (broaden) the cavity. In light of the previously discussed naturally occurring H^170^ → L mutation is was particularly interesting to test F^168^ which, like H^170^, is located within EL2 at the entrance to the binding cavity, and, like H^170^, it affects ligand binding, although apparently to a lesser extent. This provided further evidence of the role of this region in receptor functional activity.

**Table 3:** **Site-directed mutants of adrenocorticotropic hormone receptor (ACTHR) and their effects on receptor function**.

Mutation	Binding (×wtACTHR)	Cyclic adenosine 3′,5′-monophosphate (×wtACTHR)	Membrane trafficking (×wtACTHR)	Rate of internalization (% of initial rate)	Reference
D^70^ → A	X	X	X		(146)	
	
*K^77^ → A	*K*_i_ = 4.5×↑	EC_50_ = 7.8×↑	1.4×↓		
	
E^80^ → A	*K*_i_ = 11.6×↑	EC_50_ = 9.8×↑	1.3×↓		
	
*N^81^ → A	*K*_i_ = 1.8×↑	EC_50_ = 2.4×↑	1.1×↓		
	
D^103^ → N	*K*_i_ = 2.8×↑	EC_50_ = 7.3×↑	1.5×↓		
	
*D^104^ → N	*K*_i_ = 10.2×↑	EC_50_ = 18.3×↑	1.3×↓		
	
D^107^ → N	*K*_i_ > 178.6×↑	EC_50_ > 1250.0×↑	1.5×↓		
	
*F^110^ → A	*K*_i_ > 116.3×↑	EC_50_ > 208.3×↑	1.5×↓		
	
*T^164^ → A	*K*_i_ = 1.2×↓	EC_50_ = 1.3×↓	1.1×↓		
	
*F^168^ → A	*K*_i_ = 25.3×↑	EC_50_ = 68.3×↑	1.8×↓		
	
*F^178^ → A	*K*_i_ > 178.6×↑	EC_50_ > 1250.0×↑	1.5×↓		
	
F^235^ → A	*K*_i_ = 17.5×↑	EC_50_ = 270.0×↑	1.2×↓		
	
H^238^ → A	*K*_i_ > 178.6×↑	EC_50_ > 1250.0×↑	1.4×↓		
	
*F^244^ → A	*K*_i_ = 1×↔	EC_50_ = 1.1×↑	1.1×↓		
	
F^258^ → A	*K*_i_ = 13.9×↑	EC_50_ = 233.8×↑	1.3×↓		
	
D^272^ → A	X	X	X		

N^12^N^13^ → QQ		EC_50_ = ~1.4×↑	↔		(194)	
	
N^17^N^18^ → QQ		EC_50_ = ~2.1×↑	↔		
	
N^12^N^13^N^17^N^18^ → QQQQ		EC_50_ = ~4.5×↑	~3.2×↓		

wtACTHR		*R*_max_ = 1×↔	1×↔	33.1	(86)	
	
T^131^ → A		*R*_max_ = 1.1×↓	1.27×↑	<0.1	
	
T^131^ → D		*R*_max_ = 4.5×↓	1.3×↑	<0.1	
	
S^140^ → A		*R*_max_ = 1.1×↓	1.1×↓	25.5	
	
S^140^ → D		*R*_max_ = 1.2×↓	1.25×↑	43.2	
	
T^143^ → A		*R*_max_ = 33.3×↓	100×↓	22.2	
	
T^143^ → D		*R*_max_ = 1×↔	3.1×↓	16.3	
	
T^143^ → S		*R*_max_ = 1.1×↑	2.3×↓	27.7	
	
T^143^ → G		X	11.1x↓	X	
	
T^143^ → K		*R*_max_ = 2.9×↓	12.5×↓	X	
	
T^147^ → A		*R*_max_ = 1.2×↑	1.2×↑	23.6	
	
T^147^ → D		*R*_max_ = > 100×↓	>100×↓	<0.1	
	
S^202^ → A		*R*_max_ = 1×↔	1.5×↑	40.6	
	
S^202^ → D		*R*_max_ = 1.1×↑	1.1×↓	60.7	
	
S^204^ → A		*R*_max_ = 1.2×↑	1×↔	18.8	
	
S^204^ → D		*R*_max_ = 1×↔	1.5×↓	39.4	
	
S^208^ → A		*R*_max_ = 1.1×↑	1.1×↓	22.6	
	
S^208^ → D		*R*_max_ = 1.1×↓	1.1×↓	56.7	
	
T^209^ → A		*R*_max_ = 1.1×↑	1×↔	32.4	
	
T^209^ → D		*R*_max_ = 1.1×↓	1.2×↓	20.1	
	
S^280^ → A		*R*_max_ = 1×↔	1.4×↓	50.8	
	
S^280^ → D		*R*_max_ = 1.5×↓	1.1×↓	<0.1	
	
S^294^ → A		*R*_max_ = 1×↔	1.3×↓	36.4	
	
S^294^ → D		*R*_max_ = 1.4×↓	1.3×↓	49.0	
	
N^12^N^13^N^17^N^18^ → QQQQ		*R*_max_ = 1.2×↓	3.0×↓	80.5	

*×, fold difference compared with wtACTHR; ↔, value unaffected; ↑, value increased; ↓, value decreased; X, not determined; empty field, no data available*.

Although very informative, this work by Yang and colleagues was not their first involving ACTHR. In their preceding study (published only a few months earlier), they used chimeric MC4/ACTHR receptors to probe TM domain residues of MC4R that are responsible for the high affinity for the synthetic ligand NDP-MSH (195). Due to the high ligand recognition specificity, they intended to use the TM domains of ACTHR for loss of function studies, and in order to localize the alterations and simplify data interpretation, they replaced only one TM domain (TM2, TM3, TM4, TM5, or TM6) at a time without disruption of adjacent intracellular or ELs. The results revealed that in majority of cases these replacements had little or no effect on receptor surface expression, NDP-MSH recognition or receptor activation efficiency. The exception was the replacement of TM3 (hMC4R/TM3 hMC2R); although this receptor was transported to the cell surface, it displayed significantly reduced ligand affinity and potency (a 12.7-fold and 40-fold decrease, respectively; Table S1 in Supplementary Material). Therefore, the authors concluded that non-conserved residues in this region determine the ability of MC4R to effectively bind NDP-MSH (195). However, based on the above knowledge, we believe that these observations are more likely due to peculiarities of ligand recognition within ACTHR rather than MC4R, in particular binding pocket disturbance caused by the presence of an additional acidic (D^104^) residue, and also possibly the aromatic F^110^ residue (195).

The successful application of a chimeric receptor strategy for various MCRs has been reported on multiple occasions (195–198). Since this initial work number of groups including our own group has used this platform for functional research on ACTHR (199–202). However, since the results of these studies are to a degree speculative, and because different analytical methods were employed, the findings are quite difficult to summarize and so are reviewed in chronological order.

Although many groups were working in parallel, we were the first group to publish in this area. During the initial stages of our research (199), we were more interested in exploring the structural elements that determine the selectivity of ACTHR membrane expression rather than its ligand recognition selectivity. Therefore, since it was already known that posttranslational modification of the N-terminus affected the membrane transport of cell-surface glycoproteins, in our first set of chimeric receptors, we substituted this region in both ACTHR and MC4R. The results partially confirmed our hypothesis; the membrane expression of MC4R with the ACTHR N-terminus (Ch1 in Table S1 in Supplementary Material) was significantly reduced compared with wt MC4R. The results also demonstrated that the N-terminus is not the only region of ACTHR that facilitates its intracellular retention, because ACTHR with the N-terminus of MC4R (Ch2 in Table S1 in Supplementary Material) was not trafficked to the cell membrane. Thus, to explore further, we created additional sets of chimeric receptors by replacing ACTHR TM domains with corresponding regions of MC4R in various combinations (Ch3–Ch15 in Table S1 in Supplementary Material). Subsequent evaluation of the membrane transportation efficiency revealed that retention occurred only when both TM3 and TM4 from ACTHR were present simultaneously, suggesting this region, or elements of this region, is responsible for intracellular retention. In addition to membrane transportation, we also carried out binding and cAMP response analyses. Although, due to their sensitivity, the initial purpose of these assays was to enable the detection of low-level membrane trafficking, the results also identified regions that determine ACTHR ligand-binding specificity. MC4R-based chimeric receptors were able to bind ligands when TM4–5 and TM6–7 were replaced with the corresponding parts of ACTHR, but binding was not observed when both regions were replaced simultaneously. Thus, we speculated that there must be some kind of interaction between these two elements, which disrupts the conformation required for formation of the MC consensus pharmacophore (M–X–H–F–R–W) binding pocket (199).

A year after publication of our report, Hinkle and colleagues reported on employment of remarkably similar strategy (201). Indeed, several of the chimeric receptors were identical in spite of being independently created. However, they employed distinct methods for characterization of the chimeric receptor properties (i.e., measurement of surface transport and activation efficiency), and they also evaluated the effects of co-expression with MRAP1. As in our study, the chimeric receptors were generated in sets. The first set included ACTHR-based chimeric receptors with a variety of TM domains replaced to corresponding regions from MC4R (2C1–2C6 in Table S1 in Supplementary Material), while the second set mirrored these so that the same TM domains within MC4R were replaced with the corresponding regions from ACTHR (4C1–4C6 in Table S1 in Supplementary Material). Functional analysis confirmed that co-expression with MRAP1 promoted the cell-surface trafficking of ACTHR-based chimeric receptors, but this was decreased with MC4R-based receptors. However, the ACTHR-based receptor with only the N-terminus, TM1 domain, and IL1 replaced to the corresponding regions of MC4R (2C1 in Table S1 in Supplementary Material) was an exception, since it was located on the cell surface even in the absence of MRAP1, but despite its successful transportation it was functionally inactive. To investigate this further, Hinkle and colleagues created an additional set of chimeric receptors (C2C1a, C2C1b, and C2C1c in Table S1 in Supplementary Material), and subsequent evaluation of their properties narrowed the region responsible for intracellular retention to TM1 alone, because the corresponding receptor variant (C2C1b in Table S1 in Supplementary Material) was both effectively transported to the cell plasma membrane, and functionally active in the presence of MRAP1. Functional analysis of receptors from the main set also revealed another surprising discovery; when co-expressed with MRAP1, ACTHR-based chimeric receptor with TM2, EL1, and TM3 replaced to the corresponding regions of MC4R (2C2 in Table S1 in Supplementary Material) was able to induce intracellular response upon stimulation with both NDP-MSH and ACTH and also displayed substantial constitutive activity. Since both of these traits are known to be characteristic of MC4R, the authors attempted to pinpoint the responsible region by undertaking the creation of another set of C2C2 derivative chimeric receptors (C2C2a, C2C2b, C2C2c, and C2C2d in Table S1 in Supplementary Material), but functional data showed that none shared the properties of the parental chimeric or wt receptors. Based on this and other studies, Hinkle and colleagues proposed that a more likely explanation for these observations was the general misfolding of ACTHR that, without the assistance of MRAP1, is unable to pass the rigorous quality control system in the ER and is subsequently degraded. Regarding our article, the authors simply remarked that although they did not study exactly the same chimeras, but according to their data introduction of the TM2–TM3 or TM4–TM5 regions of ACTHR receptor had little effect on MC4R surface expression (201).

**Figure 7:**
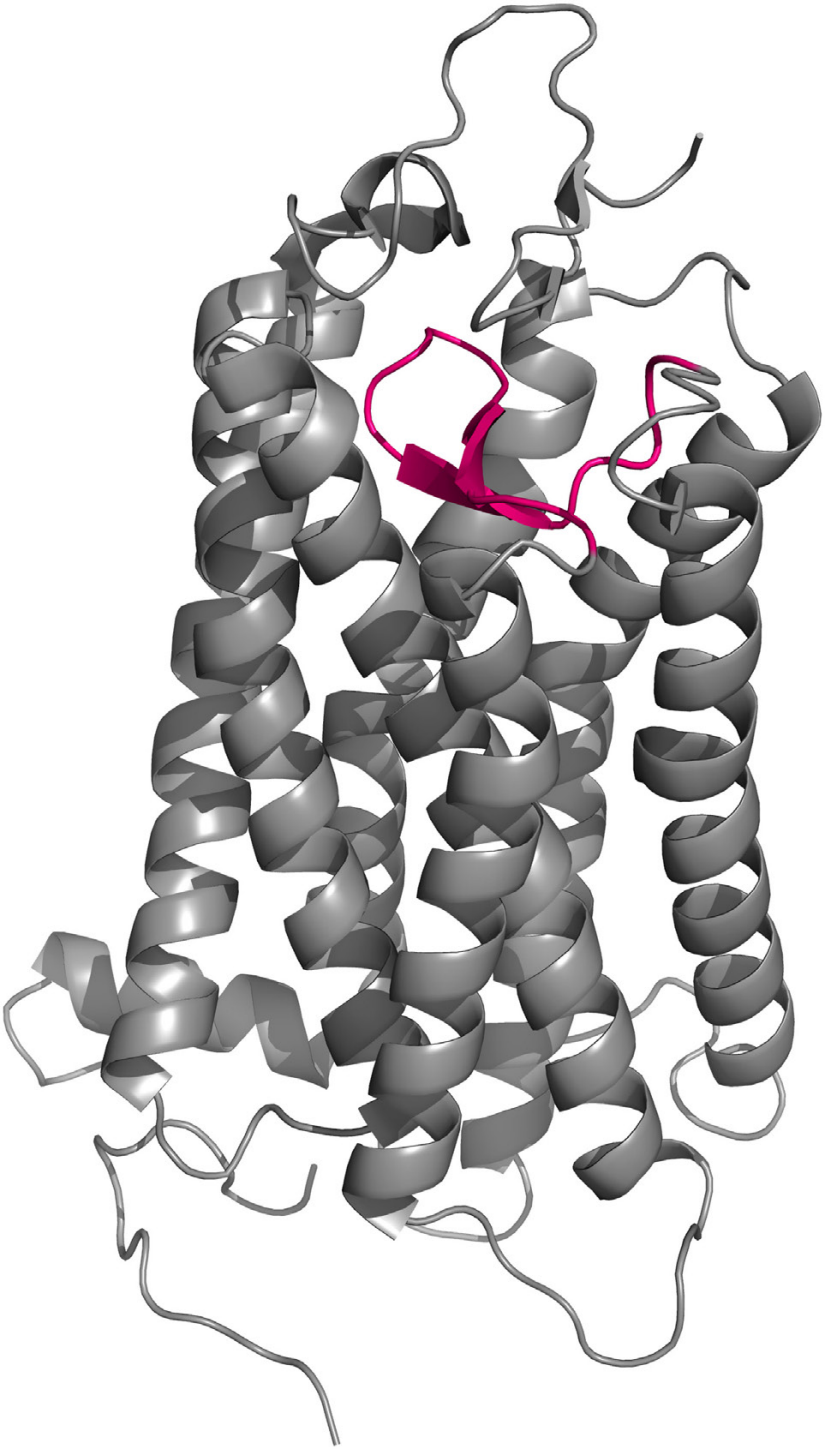
**Cartoon representation of the crystal structure of bovine rhodopsin (150)**. EL2 (magenta) forms a lid-like structure over the ligand-binding pocket that prevents any external molecular interference with the covalently bound ligand.

Based on confidence in our own results and given that the action of ACTHR remained largely unexplained, we decided to further investigate the matter. In our following study (200), we generated additional sets of ACTHR-based chimerical receptors. Given our previously proposed hypothesis that the N-terminus and TM3–TM5 region of ACTHR was important for receptor transportation and ligand recognition specificity, we replaced various elements of these regions with the corresponding elements of MC4R (Ch16–Ch22 in Table S1 in Supplementary Material). Similarly to the Hinkle group, we tested all receptors for their ability to reach the cell plasma membrane and induce intracellular cAMP responses upon stimulation with ACTH and α-MSH during both standalone expression and co-expression with MRAP. The results were somewhat unexpected because they indicated that the integrity of the extracellular part of receptor and not the separate TM domains was of paramount importance for the formation of ACTHR-specific and MRAP1-dependent arrest signal. Although initially these observations seemed to conflict with the conclusions of our former publication, in-depth reanalysis of data from both studies revealed a good agreement. In most cases, when the N-terminus, EL1 and EL2 of ACTHR were present the chimera was retained, but when two of the three elements were present, transport of the chimera to the membrane was hampered (Table S1 in Supplementary Material). Intrigued by this finding, we decided to verify and investigate it in more detail by making smaller (two to five aa) replacements within the TM3–TM5 region of Ch2, a chimera from our previous study, which is essentially ACTHR with the N-terminus of MC4R (Table S1 in Supplementary Material). While performing the same analyses as carried out on previous sets of receptors, we observed that in most cases these replacements increased the membrane export efficiency during standalone expression, but replacement of residues located within the central part of TM3 and the intracellular part of TM4 had no effect (Ch24, Ch27, and Ch28 in Figure S1 in Supplementary Material). Co-expression with MRAP1 revealed a whole spectrum of effects, including receptors that were unaffected by MRAP1 and still retained (Ch28 in Figure S1 in Supplementary Material), receptors unaffected by MRAP1 and still effectively transported to the membrane (Ch26, Ch32, and Ch35 in Figure S1 in Supplementary Material), receptors with improved transportation efficiency comparable with ACTHR (Ch24, Ch27, Ch30, Ch31, and Ch34 in Figure S1 in Supplementary Material), and receptors with decreased transportation efficiency comparable to MC4R (Ch23, Ch25, Ch29, and Ch33 in Figure S1 in Supplementary Material). As observed with large-scale replacements, these results were unexpected because we believed we finally understood ACTHR specificity. Nevertheless, like Hinkle and colleagues, we came to the conclusion that the overall structure of ACTHR must be somewhat attuned for misfolding as changes within various regions could avert this effect. Thus, we speculated that similarly to bovine rhodopsin, the extracellular part of ACTHR might form a lid-like structure over the ligand-binding pocket (Figure 7), but rather than being permanent like in rhodopsin, it must be able to undergo MRAP1- and –K–K–R–R–P– pharmacophore-induced conformational changes that result in opening of the –M–X–H–F–R–W– binding cavity. In addition, this lid-like structure could also serve as an arrest signal, since being located on the surface of the cell membrane, it is readily accessible to components of the ER quality control system. However, it also appeared that the correct formation of this structure requires a precise alignment of the TM domains, as even the slightest changes were able to disrupt its formation. Although this study, like the previous one, mainly focused on identification of mechanisms underlying the ACTHR membrane transportation specificity, we were also able to gain some insight regarding structures that determine its ligand recognition specificity. Since all chimeric receptors created during this study were ACTHR based, it was not surprising that the majority of them were able to induce cAMP responses only when co-expressed with MRAP1 and stimulated with ACTH; therefore, the most interesting in this respect were the functionally inactive Ch24 and Ch31 (Figure S1 in Supplementary Material). The functional inactivity of Ch24 was rather easy to explain, since all affected residues were located in close proximity to residues known to form the –M–X–H–F–R–W– acidic-binding pocket. However, residues replaced within Ch31 were far more interesting because they are located within EL2 that forms the edge of the binding cavity, as described above regarding the naturally occurring H^170^ → L mutation. Since none of the adjacent alterations displayed a similar effect, and because selected residues were substituted with corresponding ones from MC4R, we concluded that this region must be either involved in the formation of the –K–K–R–R– binding pocket or functional interaction between ACTHR and MRAP1 that mediates receptor activation. In an attempt to explain this observation, we also compared the properties of the original and substituted residues, which showed that H^170^ and H^171^ (substituted with D and S, respectively) were the most plausible candidates for residues with a functional role because their replacement had the most significant effect on properties of this region, and due to its potential positive charge, propensity for hydrogen-bonding, and aromatic nature, histidine is often a functionally important residue (200).

The latest in a series of publications on chimeric ACTH/MC4 receptors was published by Yang and colleagues (202). This study assessed the binding affinity and potency of the synthetic ligand [d-Phe7]ACTH(1–24) on chimera receptors that were very similar to those described in their previous report in 2007 (195) (Table S1 in Supplementary Material). Their main conclusion was also very similar to their former conclusion that TM3 of ACTHR is critical for ligand selectivity and potency. Therefore, due to their lack of novelty, these results are not reviewed in detail, although there is one aspect that must be mentioned. In this article, the authors also described the creation of five ACTHR-based receptors with single TM domain substitutions to corresponding regions of MC4R (TM2, TM3, TM4, TM5, or TM6; Table S1 in Supplementary Material). In the experimental procedures section, the authors indicated that expression of chimeric receptors was performed in both OS3 and HEK cell lines, the former of which is known to support functional expression of ACTHR. However, during the experiments, they observed that all ACTHR-based chimeric receptors were transported to the cell surface at very low levels, and they therefore failed to assess their ligand-binding affinity and potency (202). Although co-expression with MRAP1 in HEK293 or CHO cell lines and the OS3 cell line may not be directly comparable, in our view, these results are somewhat contrary to the observation made by us and the Hinkle group that most ACTHR-based chimeras co-expressed of with MRAP1 were able to reach the cell membrane and many were functionally active.

Interpreting the results of studies using ACTH/MC4 chimera receptors can be difficult and even controversial. We believe this is due to using a small-scale approach such as few chimeric receptors with single domain replacements to investigate large-scale problems such as the molecular structure and intermolecular interactions of ACTHR (i.e., overinterpretation of results acquired using the reductionist approach). This could be compared with observing the construction of a building through small holes in the fence that surrounds it. Since each group generates their own hypotheses and is only able to generate and test a limited number of mutant receptors, they see only a small part of the “bigger picture” and thus were viewing the problems from a different perspective. Nevertheless, in spite of these differences, we have attempted to summarize the disparate results in Table S1 in Supplementary Material, and, to our surprise, data obtained by different groups are generally in good agreement. Of course, some seemingly identical receptors displayed very different properties in the hands of different research groups, and differences in the expression systems may account for the apparent discrepancies (for example, Ch5 or Ch6 in our study and 2C6 or 4C4 in the Hinkle group). However, the overall general agreement allowed us to draw some summative conclusions. Firstly, it appears there is no single domain that is responsible for ACTHR intracellular retention, but rather the general structure of ACTHR may be somewhat misfolded and coupling with MRAP1 is required to either correct or mask this. Secondly, the results indicated that the role of the extracellular and possibly even the intracellular parts of ACTHR had been underestimated, because in multiple cases the substitution of even a single loop or terminus was crucial on determining receptor localization and the ability to induce an intracellular response.

While ourselves and others were working on large-scale replacements and searching for domains and regions that determine the membrane transportation and ligand recognition specificity of ACTHR, a group led by Gallo-Payet was taking a more focused approach on ACTHR glycosylation (194) and phosphorylation (86) and their effects on receptor functionality. These simple, elegant studies are reviewed below, again in chronological order.

It has been known for quite some time that glycosylation of cell-surface proteins is often required for their successful transport to the plasma membrane (203, 204). In the case of GPCRs, however, the effects of glycosylation are wider and range from being important for correct receptor folding and maturation, to having no apparent function (205–208). Prediction of potential glycosylation sites within ACTHR using such online tools as NetNGlyc 1.0 Server^1^ or GlycoEP^2^ identifies two glycosylation sites within the N-terminus of the receptor: N^12^N^13^T^14^ and N^17^N^18^S^19^ (Figure 6). Since this aspect of ACTHR functionality had not been previously researched in detail, Roy et al. (194) employed site-directed mutagenesis to eliminate these sites and performed functional expression and characterization of the resultant mutated ACTHRs within the HEK293/FRT cell line to evaluate the role of glycosylation. Upon first reading, the results of this study seemed rather confusing, as the authors observed that during standalone expression abolishment of one glycosylation site had a moderate effect on the surface transportation of ACTHR, while abolishment of both significantly reduced it. In addition, co-expression with MRAP1 (α, β, or C-terminally truncated) resulted in the effective rescue of surface expression for mutants with only one valid glycosylation site, and moderate rescue for mutants with no glycosylation sites (Table 3). The confusing part, of course, was the presence of ACTHR at the cell membrane in the absence MRAP. However, this was later explained by the discovery that this cell line actually expresses low levels of MRAP2 that promotes ACTHR membrane transportation. Although at the first glance the selection of this expression system might seem unfortunate, it actually allowed the researchers to observe the effects of glycosylation in detail because, due to overexpression and MRAP1 rescue effects, the employment of any other MRAP2-less cell line most probably would not have allowed them to distinguish between unhampered and moderately hampered transportation efficiency. The causal reason for the endogenous expression of this accessory protein could be related to the source of the particular human embryonic kidney cell line. Based on MRAP2 expression data available at “The Human Protein Atlas”^3^ (209, 210), adult kidney tissue expresses this protein at low levels. Although the same database suggests the parent (HEK293) cell line does not express this protein, it is plausible that this cell line is prone to triggering expression during later passages, hence its use in ACTHR surface expression studies should be carefully considered. In addition to cell-surface transportation measurements, the authors also performed receptor activation experiments, and all mutated receptors were functionally active, but there was a tendency for EC_50_ values to increase with the number of mutated glycosylation sites, thus it was concluded that N-glycosylation of ACTHR is not critical but does have a slight influence on receptor activity (Table 3) (194).

Another type of posttranslational modification that often plays a major role in protein functionality is phosphorylation. In the case of GPCRs, it is usually associated with receptor desensitization and internalization, and it is performed by a group of serine/threonine protein kinases known as G-protein-coupled receptor kinases (211–215). Since this aspect of ACTHR action was not previously researched in detail, Roy and coworkers (86) introduced point mutations that altered all S and T residues located within the intracellular part of the receptor to the non-phosphorylatable residue A, or to the negatively charged D that can mimic phosphorylation. Additionally, T^143^ was also mutated to S, G, and K because, as already mentioned above, the homologous position (T^157^) within MC1R is critical for receptor export to the plasma membrane and thus functional activity (86, 187, 188). Detailed activation, surface expression, and receptor internalization analyses both standalone and during co-expression with MRAP1β in the HEK293/FRT cell line generated a large number of results, the most relevant of which (in the context of this review) are presented in Table 3. The authors initially evaluated the internalization of WT ACTHR and established that ligand binding is a necessary prerequisite for ACTHR internalization. Additionally, MRAP1 was internalized along with ACTHR, further confirming that a functional receptor is formed following the formation of ACTHR and MRAP1 complex. They also revealed that stimulation with low concentrations of ACTH (<0.3 nM) did not induce any apparent internalization, and ~28% of internalized receptor molecules were recycled back to the cell surface. Having established a baseline and confirmed the necessity of further investigations on the role of intracellular S/T residues, Roy et al. (86) carried out similar analyses on all mutated receptors, and the results showed that T^143^ → A and T^147^ → D replacements disrupted transportation to the cell surface and abolished the ability to respond to stimulation with ACTH, while the internalization of receptors carrying mutations T^131^ → A, T^131^ → D, and S^280^ → D was essentially undetectable. Thus, it was concluded that these four residues (T^131^, T^143^, T^147^, and S^280^) are crucial for ACTHR expression and functional regulation (86).

## Conclusion

Data presented in this review are rather voluminous and reflect the heterogeneity of available information and applied research methodology. However, despite the difficulties, we hope that condensing this large body of work has proved useful and shall provide some novel insight. At the beginning of this article, we implied that functional ACTHR was discovered only in 2005 by Metherell et al. (39), while 1992 marks the discovery of the main subunit (38). This statement is clearly at odds with the currently used nomenclature, and the name ACTHR is even more at odds (the HGNC accepted name is MC2R). But, through this review, we have come to the conclusion that using term “receptor” is inappropriate because without MRAP1 ACTHR/MC2R does not bind to its ligand. Thus, while in this article we have ignored this, we propose that the name ACTHR should not be abandoned entirely but rather used to describe the molecular complex that specifically binds only ACTH (ACTHR-2 × MRAP1).

## Author Contributions

Preparation of this article was a joint effort of all authors. DF was coordinating the process of manuscript preparation.

## Conflict of Interest Statement

The authors declare that the research was conducted in the absence of any commercial or financial relationships that could be construed as a potential conflict of interest.

## Footnotes

^1^http://www.cbs.dtu.dk/services/NetNGlyc/.

^2^http://www.imtech.res.in/raghava/glycoep/.

^3^http://www.proteinatlas.org/.
