# Supplementary material for: ACTH Receptor (MC2R) Specificity: What Do We Know About Underlying Molecular Mechanisms?
Source: Front Endocrinol (Lausanne). 2017 Feb 6;8:13. doi: 10.3389/fendo.2017.00013 (PMC5292628; doi:10.3389/fendo.2017.00013)
Supplement: Supplementary file 1 [file Image_1.PDF]

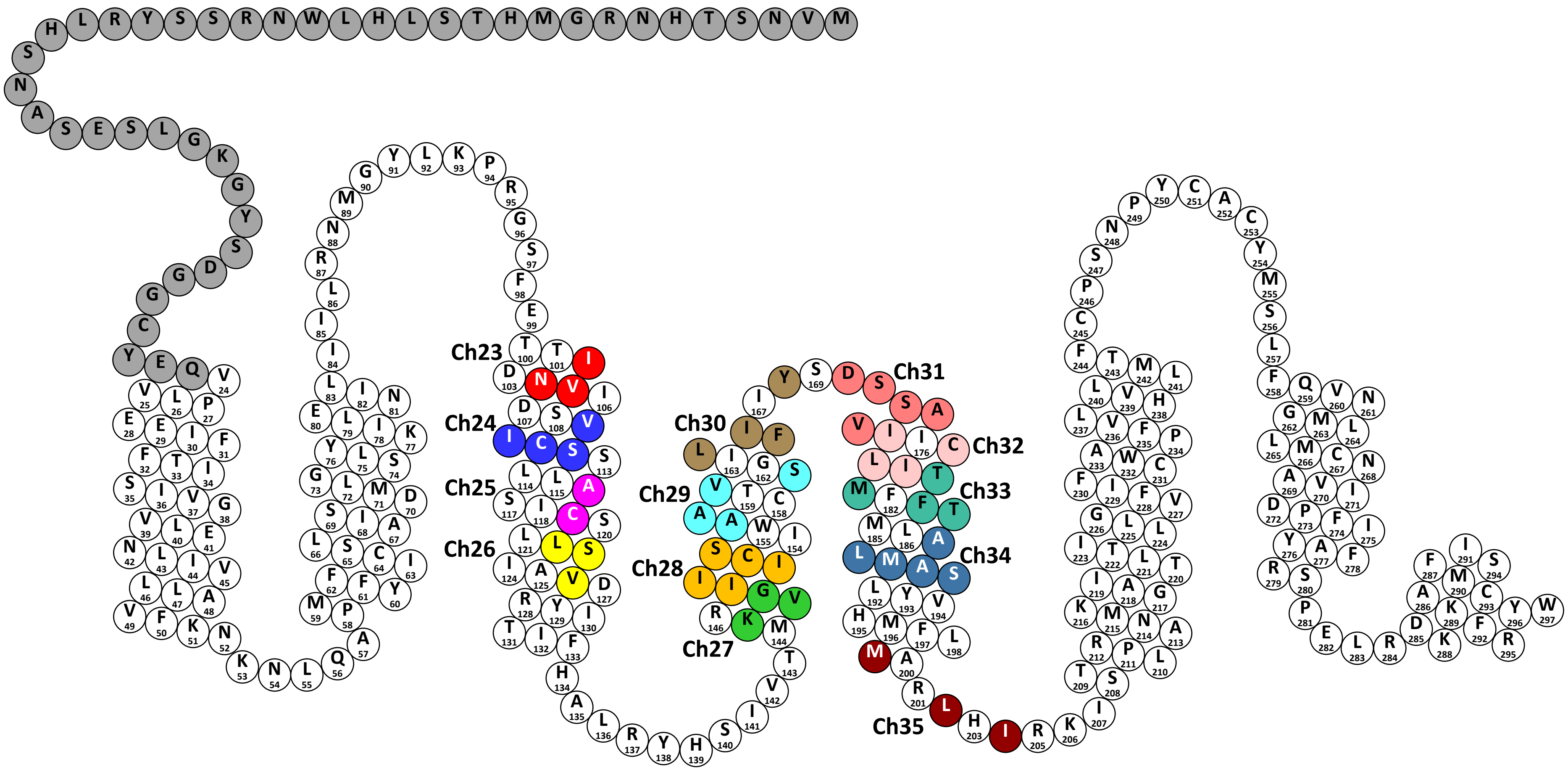

Supplementary Figure 1. Snake-like plot of recombinant receptors Ch23–Ch35 from Fridmanis et.al [1].

[1] D. Fridmanis, R. Petrovska, D. Pjanova, H.B. Schioth, and J. Klovins, Replacement of short segments within transmembrane domains of MC2R disrupts retention signal. J Mol Endocrinol 53 (2014) 201-15.
