# Supplementary material for: ACTH Receptor (MC2R) Specificity: What Do We Know About Underlying Molecular Mechanisms?
Source: Front Endocrinol (Lausanne). 2017 Feb 6;8:13. doi: 10.3389/fendo.2017.00013 (PMC5292628; doi:10.3389/fendo.2017.00013)
Supplement: Supplementary file 2 [file Table_1.DOCX]

**Supplementary Table 1.** **Chimeric ACTH/MC4 receptors and their functional properties**

| Receptor | | -MRAP | | | +MRAP | | | Reference |
| --- | --- | --- | --- | --- | --- | --- | --- | --- |
|  |  | Surface | ACTH | MSH | Surface | ACTH | MSH |  |
| MC4R |  | **++** | 1 | 1 | **+** | 1 | 1 |  |
| ACTHR |  | **-** | 0 | 0 | **++** | 1 | 0 |  |
| Ch1 |  | **+** | ^EC50^×38.1_MC4R_ | ^EC50^×7.0_MC4R_  ^Ki^×1.2_MC4R_ |  |  |  | [1; 2] |
| Ch2 |  | **-** | ^EC50^ND | ^EC50^ND | **+** | ^EC50^×129.1_ACTHR_  ^EC50^×8.0_MC4R_ | ND | [1] |
| 2C2a |  | **-** |  |  | **+** | ^RlucMax^×5.4_ACTHR_  ^RlucMax^×5.0_MC4R_ | ^RlucMax^×130.0_MC4R_ | [3] |
| 2C1c |  | **+** |  |  | **++** | ^RlucMax^×0.9_ACTHR_ |  | [3] |
| Ch3 |  | **+** | ^EC50^ND | ^EC50^ND |  |  |  | [1] |
| 2C1 |  | **+** | ^RlucMax^×ND | | **++** | ^RlucMax^×18.2_ACTHR_  ^RlucMax^×23.6_MC4R_ | | [3] |
| 2C1a |  | **+** |  |  | **++** | ^RlucMax^×1.0_ACTHR_ |  | [3] |
| 2C1b |  | **+** |  |  | **++** | ^RlucMax^×0.8_ACTHR_ |  | [3] |
| Ch4 |  | **-** | ^EC50^ND | ^EC50^ND |  |  |  | [1] |
| Ch5 |  | **++** | ^EC50^ND | ^EC50^ND |  |  |  | [1] |
| 2C6 |  | **-** | ^RlucMax^×18.2_MC4R_ | | **-** | ^RlucMax^×1.8_ACTHR_  ^RlucMax^×2.4_MC4R_ | | [3] |
| Ch6 |  | **++** | ^EC50^ND  ^Ki^×11.1_MC4R_ | ^EC50^ND  ^Ki^×0.2_MC4R_ |  |  |  | [1] |
| 4C4 |  | **-** | ^RlucMax^×12.6_MC4R_ | | **-** | ^RlucMax^×2.2_ACTHR_  ^RlucMax^×2.8_MC4R_ | | [3] |
| Ch7 |  | **+** | ^EC50^×62.3_MC4R_  ^Ki^×1.8_MC4R_ | ^EC50^×37.9_MC4R_  ^Ki^×0.7_MC4R_ |  |  |  | [1] |
| Ch8 |  | **+** | ^EC50^ND | ^EC50^ND |  |  |  | [1] |
| hMC4R/TM2 hMC2R |  |  | ^EC50^×0.04_ACTHR+MRAP_  ^Ki^×0.3_ACTHR+MRAP_  ^EC50^×1.9_MC4R_  ^Ki^×2.2_MC4R_ |  | **+** |  | ^EC50^×1.4_MC4R_  ^Ki^×2.0_MC4R_ | [4; 5] |
| Ch9 |  | **+** | ^EC50^ND | ^EC50^ND |  |  |  | [1] |
| hMC4R/TM3 hMC2R |  |  | ^EC50^×0.84_ACTHR+MRAP_  ^Ki^×1.2_ACTHR+MRAP_  ^EC50^×35.6_MC4R_  ^Ki^×9.6_MC4R_ |  | **+** |  | ^EC50^×40.0_MC4R_  ^Ki^×12.7_MC4R_ | [4; 5] |
| hMC4R/TM4 hMC2R |  |  | ^EC50^×0.04_ACTHR+MRAP_  ^Ki^×0.2_ACTHR+MRAP_  ^EC50^×1.7_MC4R_  ^Ki^×1.6_MC4R_ |  | **+** |  | ^EC50^×2.1_MC4R_  ^Ki^×1.9_MC4R_ | [4; 5] |
| hMC4R/TM5 hMC2R |  |  | ^EC50^×0.05_ACTHR+MRAP_  ^Ki^×0.2_ACTHR+MRAP_  ^EC50^×2.0_MC4R_  ^Ki^×1.3_MC4R_ |  | **+** |  | ^EC50^×1.8_MC4R_  ^Ki^×1.4_MC4R_ | [4; 5] |
| Ch10 |  | **++** | ^EC50^×124.3_MC4R_  ^Ki^×12.7_MC4R_ | ^EC50^×137.0_MC4R_  ^Ki^×3.1_MC4R_ |  |  |  | [1] |
| hMC4R/TM6 hMC2R |  |  | ^EC50^×0.04_ACTHR+MRAP_  ^Ki^×0.2_ACTHR+MRAP_  ^EC50^×1.7_MC4R_  ^Ki^×1.4_MC4R_ |  | **+** |  | ^EC50^×1.6_MC4R_  ^Ki^×1.7_MC4R_ | [4; 5] |
| 4C2 |  | **++** | ^RlucMax^×ND | | **+** | ^RlucMax^×ND | | [3] |
| Ch11 |  | **++** | ^EC50^ND  ^Ki^×2.5_MC4R_ | ^EC50^ND  ^Ki^×0.5_MC4R_ |  |  |  | [1] |
| 4C3 |  | **++** | ^RlucMax^×1.4_MC4R_ | | **+** | ^RlucMax^×0.9_ACTHR_  ^RlucMax^×1.2_MC4R_ | | [3] |
| 4C1 |  | **-** | ^RlucMax^×7.8_MC4R_ | | **-** | ^RlucMax^×4.8_ACTHR_  ^RlucMax^×6.2_MC4R_ | | [3] |
| Ch12 |  | **+** | ^EC50^ND | ^EC50^ND |  |  |  | [1] |
| Ch14 |  | **++** | ^EC50^ND  ^Ki^×0.5_MC4R_ | ^EC50^ND  ^Ki^×1.0_MC4R_ |  |  |  | [1] |
| 4C6 |  | **+** | ^RlucMax^×ND | | **-** | ^RlucMax^×18.4_ACTHR_  ^RlucMax^×23.9_MC4R_ | | [3] |
| Ch13 |  | **-** | ^EC50^ND | ^EC50^ND |  |  |  | [1] |
| Ch15 |  | **+** | ^EC50^×4.0_MC4R_  ^K^×^i^0.3_MC4R_ | ^EC50^×1.7_MC4R_  ^Ki^×0.3_MC4R_ |  |  |  | [1] |
| 2C4 |  | **+** | ^RlucMax^×ND | | **+** | ^RlucMax^×ND | | [3] |
| 4C5 |  | **-** | ^RlucMax^×25.1_MC4R_ | | **+** | ^RlucMax^×ND | | [3] |
| 2C2 |  | **-** | ^RlucMax^×4.0_MC4R_ | | **+** | ^RlucMax^×2.1_ACTHR_  ^RlucMax^×1.9_MC4R_ | ^RlucMax^×1.9_MC4R_ | [3] |
|  |  |  |  |  |  | ^RlucMax^×1.7_ACTHR_  ^RlucMax^×2.3_MC4R_ | |  |
| Ch16 |  | **+** | ^EC50^ND | ^EC50^ND | **-** | ^EC50^×4.5_ACTHR_  ^EC50^×0.3_MC4R_ | ^EC50^ND | [2] |
| Ch19 |  | **++** | ^EC50^ND | ^EC50^ND | **++** | ^EC50^×239.3_ACTHR_  ^EC50^×14.9_MC4R_ | ^EC50^ND | [2] |
| 2C3 |  | **+** | ^RlucMax^ND | | **++** | ^RlucMax^×1.0_ACTHR_  ^RlucMax^×1.3_MC4R_ | | [3] |
| hMC2R/TM4 hMC4R |  | **-** | ^EC50^ND | ^EC50^ND | **-** | ^EC50^ND | ^EC50^ND | [5] |
| Ch20 |  | **++** | ^EC50^ND | ^EC50^ND | **++** | ^EC50^×16.2_ACTHR_  ^EC50^×1.0_MC4R_ | ^EC50^ND | [2] |
| Ch21 |  | **++** | ^EC50^ND | ^EC50^ND | **-** | ^EC50^ND | ^EC50^ND | [2] |
| Ch17 |  | **++** | ^EC50^ND | ^EC50^ND | **+** | ^EC50^×5.3_ACTHR_  ^EC50^×0.3_MC4R_ | ^EC50^ND | [2] |
| Ch18 |  | **-** | ^EC50^ND | ^EC50^ND | **-** | ^EC50^ND | ^EC50^ND | [2] |
| hMC2R/TM5 hMC4R |  | **-** | ^EC50^ND | ^EC50^ND | **-** | ^EC50^ND | ^EC50^ND | [5] |
| hMC2R/TM6 hMC4R |  | **-** | ^EC50^ND | ^EC50^ND | **-** | ^EC50^ND | ^EC50^ND | [5] |
| Ch22 |  | **-** | ^EC50^ND | ^EC50^ND | **++** | ^EC50^ND | ^EC50^ND | [2] |
| hMC2R/TM3 hMC4R |  | **-** | ^EC50^ND | ^EC50^ND | **-** | ^EC50^ND | ^EC50^ND | [5] |
| 2C2c |  | **-** |  |  | **++** | ^RlucMax^×4.7_ACTHR_  ^RlucMax^×4.4_MC4R_ | ^RlucMax^×43.3_MC4R_ | [3] |
| hMC2R/TM2 hMC4R |  | **-** | ^EC50^ND | ^EC50^ND | **-** | ^EC50^ND | ^EC50^ND | [5] |
| 2C2b |  | **-** |  |  | **-** | ^RlucMax^×7.3_ACTHR_  ^RlucMax^×6.7_MC4R_ | ^RlucMax^×130.0_MC4R_ | [3] |
| 2C5 |  | **-** | ^RlucMax^×6.6_MC4R_ | | **-** | ^RlucMax^×3.6_ACTHR_  ^RlucMax^×4.7_MC4R_ | | [3] |

× , fold difference; Superscript, compared values; Subscript, wild type receptor to which measurement was compared;
ND, not determined; Regions from the MC4R are shaded grey; Regions from the ACTHR are shaded black; empty field, no data available.
++, high membrane transportation; +, low membrane transportation; , no membrane transportation;
Approximate RlucMax values from Hinkle et al. [3] were acquired through digitalization of the included bar graphs.

[1] D. Fridmanis, R. Petrovska, I. Kalnina, M. Slaidina, R. Peculis, H.B. Schioth, and J. Klovins, Identification of domains responsible for specific membrane transport and ligand specificity of the ACTH receptor (MC2R). Mol Cell Endocrinol 321 (2010) 175-83.

[2] D. Fridmanis, R. Petrovska, D. Pjanova, H.B. Schioth, and J. Klovins, Replacement of short segments within transmembrane domains of MC2R disrupts retention signal. J Mol Endocrinol 53 (2014) 201-15.

[3] P.M. Hinkle, M.N. Serasinghe, A. Jakabowski, J.A. Sebag, K.R. Wilson, and C. Haskell-Luevano, Use of chimeric melanocortin-2 and -4 receptors to identify regions responsible for ligand specificity and dependence on melanocortin 2 receptor accessory protein. Eur J Pharmacol 660 (2011) 94-102.

[4] M. Chen, M. Cai, C.J. Aprahamian, K.E. Georgeson, V. Hruby, C.M. Harmon, and Y. Yang, Contribution of the conserved amino acids of the melanocortin-4 receptor in [corrected] [Nle4,D-Phe7]-alpha-melanocyte-stimulating [corrected] hormone binding and signaling. J Biol Chem 282 (2007) 21712-9.

[5] Y. Yang, V. Mishra, C.J. Crasto, M. Chen, R. Dimmitt, and C.M. Harmon, Third transmembrane domain of the adrenocorticotropic receptor is critical for ligand selectivity and potency. J Biol Chem 290 (2015) 7685-92.
